# A Community Engagement Approach to Snakebite Prevention in Rural Uganda: Exploring Knowledge, Attitudes, and Practices

**DOI:** 10.3390/toxins18020078

**Published:** 2026-02-02

**Authors:** Kevin Arbuckle, Yowasi Byaruhanga, Hazel J. Nichols, Cris M. Kaseke, Francis Mwanguhya, Jessica Mitchell

**Affiliations:** 1 Department of Biosciences, Faculty of Science and Engineering, Swansea University, Swansea SA2 8PP, UK; h.j.nichols@swansea.ac.uk; 2 New Life Junior School, Rubirizi District, Bushenyi P.O. Box 74, Uganda; yowasi@live.com; 3 Lake Katwe United Beekeepers Association (LAKASUBA) Limited, Kasese District, Kasese P.O. Box 81, Uganda; kasekesafari@gmail.com; 4 Banded Mongoose Research Project, Queen Elizabeth National Park, Kasese District, Lake Katwe P.O. Box 66, Uganda; fmwanguhyatwooki@gmail.com; 5 Global Academy for Agriculture and Food Systems, College of Medicine and Veterinary Medicine, University of Edinburgh, Roslin EH25 9RG, UK; jmitch2@ed.ac.uk

**Keywords:** snakebite, prevention strategies, KAP, behavioural change, public health interventions, community engagement

## Abstract

Snakebite envenoming is classified as a Neglected Tropical Disease and causes mortality, morbidity, and economic impacts for hundreds of thousands of people per year, particularly in tropical, low- and middle-income countries. Most research on snakebite interventions focuses on improving clinical management rather than bite prevention. However, prevention may provide a better mechanism to minimise snakebite impacts, particularly in rural areas where access to effective medical treatment is limited. This study reports on the preliminary testing phase of a participatory workshop intervention run in rural Uganda in 2022–23, which used a community engagement approach designed to reduce snakebites through discussing snake behaviour and biology. A mixed methods survey and semi-structured interviews were conducted, both with workshop attendees and non-attendees, after the delivery of the workshops. We found that a fearful attitude toward snakes often led to human–snake conflict, with snake killings occurring commonly, and some bites occurring during attempted killings. Workshops appeared to challenge negative attitudes, as understanding snake behaviour seemed to build compassion toward snakes and therefore has the potential to reduce human–snake conflict. Those who attended workshops were more likely to suggest ‘giving snakes space,’ rather than attempting to kill them, and were more likely to suggest hospital treatment if bitten. We also found that many effective methods for snakebite prevention are already known to the community, but those who attended the workshop were aware of a wider range of prevention methods and were more likely to implement less ecologically damaging and more effective strategies. This emphasises that appropriate knowledge resides within the community to prevent snakebites, and so community engagement approaches can improve prevention practices while recognising that the ownership and knowledge for such changes is generated by the local people themselves.

## 1. Introduction

Snakebite envenoming is a debilitating public health problem which globally causes up to 137,880 deaths per year [1]. It has recently been classified by the World Health Organisation (WHO) as a Neglected Tropical Disease, defined as ‘conditions which are associated with devastating health, social and economic consequences and that are mainly prevalent among impoverished communities in tropical areas, although some have a much larger geographical distribution’ [2] (near global in the case of snakebites). Aside from the mortality risk, many venomous snakebites have life-changing implications for victims who survive with severe morbidity. For instance, at least 400,000 people worldwide are left with permanent physical disability and psychological trauma following snakebite, both of which have negative consequences for the victim’s ability to sustain their livelihood and hence support themselves and their families [3,4].

Importantly, snakebites are an issue which extends beyond human health, notwithstanding the major impact in that area, and represents a One Health problem that also affects both wild and domestic animals. Although studies of snakebite on non-human animals are currently rare, a wide range of domestic species, including both pets and livestock, are bitten by snakes worldwide, often with high case fatality rates [5]. Community-based surveys in Nepal and Cameroon suggest that the incidence of snakebite on livestock is underestimated, but that in most cases, bites to livestock impose serious impacts on livelihoods, sometimes with losses greater than average monthly earnings [6]. Economic impacts can derive from direct loss of animals, reduced production as a result of sequelae from bites, and out-of-pocket costs of veterinary healthcare, even if the animal survives [7]: all especially important in rural communities who rely on livestock for food and income. The human consequences of snakebites also result in conservation problems [8]. This is because snakes are commonly killed in attempts to prevent snakebites, such that ‘persecution or control’ represents the most common threat to snakes globally, except for habitat loss [9]. Hence, snakebites pose a multifarious and integrated set of challenges for humans, domestic animals, and wild species.

While much effort has been expended to reduce the snakebite burden in countries with the most serious problems on a global scale, many countries which have relatively high rates of bites have received far less attention. For instance, Uganda is estimated to have ~14,000 snakebite envenomations each year, resulting in ~650 deaths, ~750 amputations, and ~3000 cases of post-traumatic stress disorder [10]. Notably, these are expected to be substantial underestimates [11,12], particularly since data typically are not available for Uganda, which leaves estimates based on assumptions and extrapolations from other countries [13,14,15]. Uganda is home to 26 venomous snake species of clinical importance [16], including 14 highly venomous species (category one or two [17]). Within Uganda, the greatest number of highly venomous species occur in the rural southwest of the country [18], although substantial uncertainty remains over the presence and distribution of reptile species within Uganda [19].

Estimates of travel time to hospitals are not untenably long in Uganda [18], but these are based on the assumptions that roads are good quality and transport is readily available: often, neither of which are the case [20]. Moreover, hospital supplies of antivenom are highly variable and often non-existent, and medical training for snakebites is limited [21,22,23,24]. For example, a survey of Ugandan healthcare practitioners found that >90% had never had any training in snakebite management, >75% had low knowledge in this area of medicine, and >50% recommended potentially dangerous first aid methods [22].

Given the challenges of accessing appropriate healthcare, a reduction in the burden of snakebite envenoming cannot rely on treatment alone and must instead focus strongly on prevention [25]. Indeed, a model of snakebite control measures revealed that prevention was more effective than treatment at reducing the impact of snakebite envenoming, although a combined approach is clearly necessary [26]. Nevertheless, research on the effectiveness of interventions for snakebite prevention is still severely lacking [27,28].

To prevent snakebites, one must first understand the context in which snakebites occur and why. Such context may include human behaviour and the knowledge, attitudes and practices which underpin action. It is widely accepted that knowledge alone does not lead to behavioural change [29,30,31]. Rather, people must come to their own conclusions regarding if and why an alternative behaviour is needed, and how this is feasible for them to implement. As such, top-down solutions to human–snake conflict are unlikely to reduce aggression toward snakes nor incidents of snakebite in humans [32]. An alternative approach involves bottom-up solutions whereby people are provided with space to explore the problem and suggest appropriate and actionable changes they could make to their own behaviour.

Community Engagement (CE) research is one such bottom-up solution which is becoming increasingly popular across public, global and One Health fields [33,34,35,36,37]. Within the present study we use the following definition of community engagement:

*“a participatory process through which equitable partnerships are developed with community stakeholders, who are enabled to identify, develop and implement community-led sustainable solutions using existing or available resources to issues that are of concern to them and to the wider global community.”*

[38]

A scoping review by Moos et al. [27] and other recent studies specifically highlight CE’s potential for snakebites prevention [39,40], yet neither Moos et al. [27] nor Rodrigo and Gnanathasan [28] found any publications which used and evaluated this approach as a means of preventing snakebite.

Here, we report the initial findings based on an intervention to address snakebite prevention in rural Uganda that was designed to be an effective CE research process. We explore a range of data linked to the process of preliminary testing a participatory snakebite prevention workshop. Using the data in this way is intended to allow the community voice to inform development of the workshops into a full testing phase, whilst also contributing knowledge to the poorly understood context of snake-related behaviour and bite risk in this area.

Our study aims to (1) explore the knowledge, attitudes, and practices of community members in rural Uganda around snakes and snakebites, (2) evaluate the extent to which participatory workshops can generate new knowledge of snakes and snakebite prevention strategies, and (3) assess whether such workshops have potential to create lasting change in attitudes and behaviours linked to human–snake coexistence.

## 2. Results

### 2.1. Dataset

#### 2.1.1. Workshop Observations

Seven participatory workshops were delivered to 157 community members in July 2022 and May 2023. Workshops involved an introductory discussion of snake behaviour that is relevant to snakebites avoidance, a ‘thought experiment’ to try to develop empathy and understanding of why snakes bite, identification (by community members) of the potential benefits of snakes, discussion of risks associated with attempting to kill snakes, and basic information on the proportion of local snake species that are harmful. This was followed by a walk around the local area, where community members identified areas where human–snake conflict is likely to occur and what actions might reduce this (based on previous knowledge and experience or new knowledge of snake behaviour gained via the first discussion). After a break, participants discussed potential methods of reducing snakebite and watched a WHO video on snakebite prevention and first aid. Finally, participants were given the opportunity to ask questions and give feedback on the workshop. See Section 5 for further details. Three of the workshops delivered in July 2022 were observed by a qualitative researcher.

#### 2.1.2. Study Sample

##### Survey

Between July 2022 and August 2023, a written survey was completed by 76 respondents. Due to the impacts of COVID-19, we were unable to deliver surveys pre- and post-workshop. Instead, we assessed the potential impact of the workshops through a cross-sectional approach. Respondents consisted of 25 people who had attended a snakebite workshop, eight who had not attended but had spoken to an attendee, and 43 who had neither attended nor had spoken to an attendee. Following consideration by the Uganda Research Ethics Committee at Makerere University, we did not have permission to request personal data from the survey respondents (gender, age, educational level, etc.).

##### Interviews

Between 7th and 9th May 2023, we conducted 21 semi-structured interviews with 15 adult women, aged 20–50, and six adult men, aged 18–50 (Table S8). Of the 21 interviewees, 10 had attended a workshop (approximately 10 months beforehand), 8 had not attended but had heard of the workshops by word of mouth, and 4 had not attended the workshops and had not heard of them.

#### 2.1.3. Study Approach

Using a combination of the workshop observations, surveys, and key informant interviews, we focused on evaluating the knowledge, attitudes, and practices (KAP) of rural Ugandan communities in relation to snakes, snakebite treatment, and prevention. This study took a mixed-methods approach, with numerical data being analysed quantitively, and qualitative data being reported as a narrative summary (see Section 5 for further details).

First, we (1) ascertained the knowledge of community members of the snake species that are present in the local area and (2) assessed their ability to evaluate the risks posed by different snake species. We then moved on to (3) assess attitudes towards snakes, including both positive and negative aspects, and (4) the perception of human mortality risk from snakebites. Finally, we investigated (5) the practices of participants and the wider community in relation to snakebite treatment and prevention. Unless specified otherwise, we begin each section of the results by summarising responses of community members who did not attend the workshops, and we then move on to comparing the responses of attendees and non-attendees (who had and had not spoken to attendees about the workshops). The exploratory nature of this study allows for data from the non-attenders to be considered as local snake-related knowledge, attitudes, and practices without interference from the workshop information. Data from the workshop attendees is discussed in reference to workshop content, identifying trends in knowledge gains and changes in attitudes and practice, and thus pathways-to-impact from the workshops. The non-attending participants who spoke to attendees provide initial indications of how knowledge diffuses in this community.

### 2.2. Knowledge

#### 2.2.1. Identification of Snakes by Community Members

In the survey, participants were shown 12 photographs of snakes. For each, they were asked if the snake was familiar to them, if they could name the species, and to assess whether they thought it was dangerous or harmless. Survey participants who had not attended the workshop could correctly identify the species of a mean of 1.65 (of 12) snakes shown in photographs (Table S1). There were clear trends in recognisable snake species, with the forest cobra (in a defensive posture) and brown house snake being recognised by most participants (>74%). The rock python was identified by 11.6% of participants and only one participant identified the brown forest cobra in a non-defensive position (Table S1).

When interviewed face-to-face (without the presence of reference photographs), community members could name and describe up to five snake species in their local language (Table S2). The same five snakes were named by workshop attendees, non-attendees, and those who had not attended but had spoken to people who had; the encwera (black spitting cobra), ekiryambeba (‘rat eater’ or brown house snake), empiri (puff adder), enzira mire (python), and enyarubabi (green snake). Monitor lizards (enswaswa) were considered a species of snake by three interviewees (one in each workshop attendance category), and ekirumira habiri (double or two-headed snake) was named by two interviewees (an attendee and a person who had heard of the workshops), but we were unable to identify the species that was being referred to by this name. The (Nile) crocodile was named by two attendees (an attendee and a person who had heard of the workshops), a yellow snake (species not identified) was described by one person (an attendee) and another person (a non-attendee who had not heard of the workshops) as an ensigira mutete (possibly referring to monitor lizards, but it was described as a ‘four-legged splitting snake’ which chases people, hits its head off the ground and splits in two, and produces mushrooms to eat them). The inclusion of some non-snake species is likely because *enjoka*, the local word for snake, can also be interpreted in some dialects to mean scaled animal more generally.

#### 2.2.2. Association of Workshop with Snake Identification

**Figure 1:**
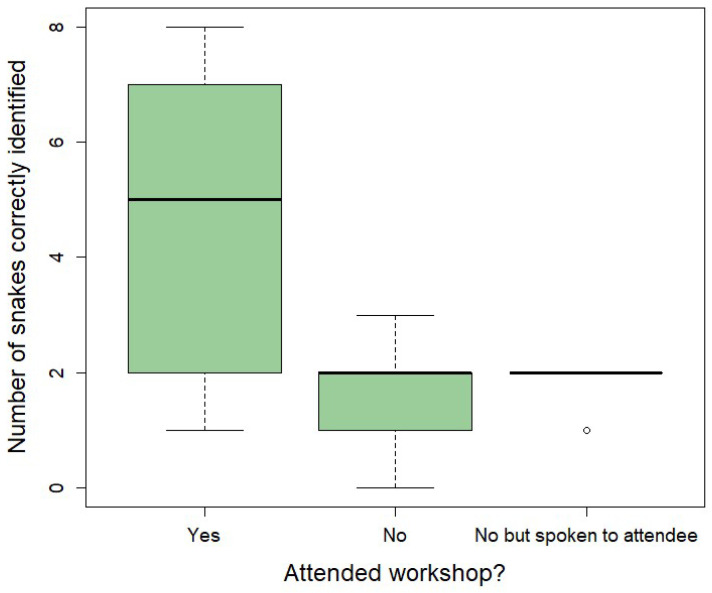
Number of snake species correctly identified from photographs by 76 survey responses (survey question 8) in relation to workshop attendance. Workshop attendees correctly identified more snake species in comparison to non-attendees.

Although workshops did not provide specific training on snake identification, our survey found that workshop attendees could correctly identify significantly more snake species from photographs (mean = 4.8 correct answers) than both non-attendees who had heard of the workshops by word of mouth (mean = 1.9 correct answers; Poisson regression coefficient = −1.059, standard error (SE) = 0.150, z = −7.060, *p* = 1.66 × 10^−12^; Figure 1) and non-attendees who had not heard of the workshops (mean = 1.7 correct answers; Poisson regression coefficient = −0.932, SE = 0.274, z = −3.400, *p* = 0.001; Figure 1). However, there was no difference in snake identification ability between non-attendees who had and had not heard of the workshop (Poisson regression coefficient = −0.127, SE = 0.284, z = −0.447, *p* = 0.655; Figure 1).

Improvements in identification skills of attendees from photographs seemed to be associated with particular snake species (Table S1). The most notable example is the puff adder; all 19 respondents who correctly identified the puff adder were workshop attendees, bar one who had not attended but had spoken to an attendee. Similarly, the majority of correct rock python identifications came from workshop attendees, and only workshop attendees correctly identified five other snake species (Table S1).

Comparatively, when interviewed face-to-face, there were no notable differences between interviewees’ ability to name or describe snake species in relation to their attendance at workshops (Table S2).

#### 2.2.3. Risk Assessment of Snakes by Community Members

In general, this community believed most snake species were dangerous, with most workshop participants vastly overestimating the proportion of local snake species that were dangerous to humans. In the survey, 8 of the 12 snakes (comprising 11 species) shown in the photographs were potentially dangerous to humans. Participants who had not attended or heard about the workshops correctly identified the level of risk of a mean of 8.42 of the 12 snakes in the images (Table S3). Of the eight dangerous snakes, the vast majority of these non-attendees correctly classified them as dangerous. However, of the four harmless snakes, three of these were also consistently classified as dangerous, with the one exception being the brown house snake, which was correctly classed as harmless by 39/43 participants (Table S3).

#### 2.2.4. Association of Workshop with Risk Assessment of Snakes

**Figure 2:**
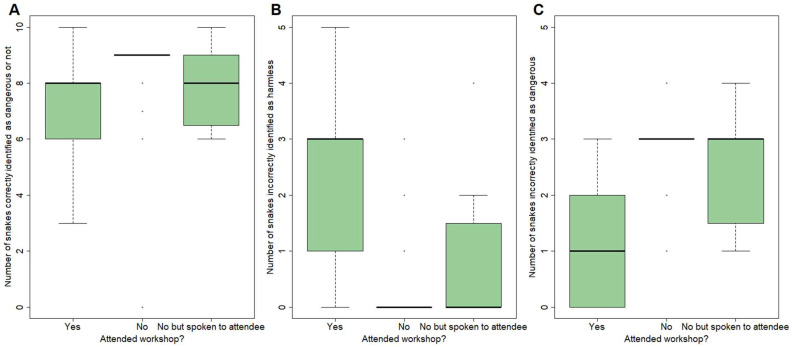
The number of snake species correctly identified as harmless or dangerous from photographs by 76 survey responses in relation to workshop attendance. (**A**) Workshop attendees had a similar number of correct answers compared to non-attendees. However, the nature of the errors showed divergent patterns, with attendees being more likely to incorrectly suggest that a snake was harmless (shown in (**B**)) and non-attendees being more likely to incorrectly suggest that a snake was dangerous (shown in (**C**)).

The number of correct risk assessments (as dangerous vs. harmless) was similar, regardless of workshop attendance (Table S3, Figure 2). Workshop attendees had a similar number of correct answers compared to non-attendees who had heard of the workshops by word of mouth (Poisson regression coefficient = 0.057, SE = 0.146, z = 0.390, *p* = 0.697) and non-attendees who had not heard of the workshops (Poisson regression coefficient = 0.124, SE = 0.090, z = 1.370, *p* = 0.171). Furthermore, there was no difference in snake risk assessment ability between non-attendees who had and had not heard of the workshop (Poisson regression coefficient = 0.067, SE = 0.137, z = 0.489, *p* = 0.625).

However, importantly, the nature of errors showed divergent patterns (Table S3, Figure 2). Specifically, workshop attendees were more likely to incorrectly consider a snake to be harmless compared to non-attendees (attendees vs. non-attendees who had not heard about the workshop, Poisson regression coefficient = −2.088, SE = 0.306, z = −6.836, *p* = 8.14 × 10^−12^; attendees vs. non-attendees who had heard about the workshops, Poisson regression coefficient = −1.026, SE = 0.399, z = −2.570, *p* = 0.010). In contrast, non-attendees were more likely to incorrectly consider a snake to be dangerous compared to attendees (attendees vs. non-attendees who had not heard about the workshops, Poisson regression coefficient = 0.852, SE = 0.201, z = 4.246, *p* = 2.17 × 10^−5^; attendees vs. non-attendees who had heard about the workshops, Poisson regression coefficient = 0.701, SE = 0.287, z = 2.445, *p* = 0.015).

Non-attendees who had spoken to attendees showed similar patterns to non-attendees who had not heard about the workshops (Table S3, Figure 2) in terms of identifying harmless snakes as dangerous (Poisson regression coefficient = 0.151, SE = 0.241, z = 0.626, *p* = 0.531). However, non-attendees who had spoken to attendees showed an intermediate level of incorrectly classifying a snake as harmless, and were significantly more likely to make this type of incorrect classification compared to non-attendees that had not spoken to an attendee (Poisson regression coefficient = −1.063, SE = 0.469, z = −2.267, *p* = 0.0234).

### 2.3. Attitude Towards Snakes

#### 2.3.1. Attitude of Community Members Towards Snakes

Within interviews and workshops, participants generally reported a negative attitude toward snakes. Workshop participants generally appeared visibly uncomfortable when asked if they liked snakes and most would immediately shake their head and say ‘no’. When asked why, they often responded with fear of bites. Indeed, within the interview data, the most common emergent theme related to attitude was a fear of bites, reported by 13 out of 21 interviewees (Table 1). This was particularly so for the 11 who did not attend a workshop.

*“Because of the bites we don’t like them. Because when we see them we feel scared.”*

Interviewee 3 (Non-attendee)

*“I don’t love them in any way because they are venomous and all snakes are venomous and when they see me they might bite me so I hate them.”*

Interviewee 18 (Non-attendee who had spoken to an attendee)

A negative attitude toward snakes appeared to translate into violence toward snakes. Six interviewees suggested that fear has resulted or does result in them hitting or killing snakes.

*“They bite us, when I see them I just get a stick and beat them up. When I see it before it sees me I get a stick and hit on the head.”*

Interviewee 16 (Non-attendee who had spoken to an attendee)

When asked about the positive or useful qualities of snakes, 4 of the 11 non-attendees (aggregating those who had and had not spoken to attendees) could not identify any benefits of snakes (Table 2). The remainder identified a range of benefits, including snakes’ ability to kill rodents, using their skins to make drum skins and other items, use as (dog) food, boosting tourism, and use in research and medicine, in addition to ‘feeling good looking at them’ (Table 2).

*“Drums are made from the python skins… shoes… bags are made from the skins of pythons and other snakes. Some of them eat rats so they protect our groundnuts and the rest.”*

Interviewee 3 (Non-attendee)

Snakes were also suggested to produce mushrooms.

*“There are snakes that produce mushrooms. That one that chases you and then makes it splits itself when you just leave it, so it’s good to make mushrooms.”*

Interviewee 21 (non-attendee who had heard about the workshops)

The mushroom-producing ability of snakes was also mentioned by two interviewees (one non-attendee and one attendee) when asked to describe snakes found in the community.

#### 2.3.2. Association of Workshop with Attitudes Towards Snakes

In interviews, workshop attendees often still reported fearing snakes but generally showed more positive views toward snakes (Table 1) and all cited the workshop content as being a driving force in their change in attitude.

*“The other time I used to think about them as killers but now from the workshop I think when given peace they can live in harmony with humans.”*

Interviewee 9 (Attendee)

In some cases, this change in perception has led to a self-reported change in behaviour, with all five attendees who previously reported hitting or killing snakes now stating that they no longer do so.

*“I used to fear snakes some time back before the workshop I would get a stick to hit on their head whenever I met them. Since the workshop I learnt that I can let them go, and since I learnt that giving them peace they have also given peace to me and I no longer panic like the other time. Though I still fear them, they are nice when you let them go.”*

Interviewee 14 (Attendee)

Within interviews, workshop attendees were also receptive to the discussions around snakes’ biological needs, such as access to water and shelter, and identified these as reasons why humans and snakes could come into contact.

*“We used to think that snakes bite them [people] but now we found out from the workshop that it [the snake] is always looking for other forms of survival like water or food, or may be shelter”*

Interviewee 10 (attendee)

Workshop attendees appeared to be more familiar with the potential benefits that snakes can bring, with all ten attendees giving at least one benefit, and most listing several (Table 2). The same benefits were given by non-attendees. Whilst three attendees listed snakes as a potential food source, they do not appear to be eaten by people in this community.

*“We hear other people eat them, so they are important as eaten as food.”*

Interviewee 10 (attendee)

#### 2.3.3. Personal Recollections of Snakebite Experience

Questions 3 and 4 of the survey asked for personal recollections of snakebites (how many bites over what time period, and how many bites resulted in death). Considering all 76 survey participants together (i.e., not splitting data by workshop attendance), all participants reported personal knowledge of snakebites in recent memory, with each individual recalling one to six (mean = 2.3) recent bites and a mean of 0.6 bites per month. Across the cohort, participants reported a total of 178 bites, 6 (3.4%) of which were believed to have resulted in death (Table 3). A notable trend is that despite an overall low experiential fatality rate from snakebite, most respondents gave high estimates of fatality rates from untreated bites by dangerous snakes, with non-attendees with no knowledge of the workshops estimating a mean fatality rate of 89% (range 20–100%, Table 3).

Similarly, all but one of the 21 interviewees had heard of a snakebite within their community (usually multiple bite incidents) and all 20 of these could describe at least one bite incident that they had directly observed. Only one bite was reported to be fatal, although several interviewees were not aware of the outcome of the bites they reported. The circumstances of bites are described in Table 4. There were no obvious gender disaggregation for bite victims, but the two reported bites whilst trying to kill a snake were both received by men.

#### 2.3.4. Association of Workshop with Perception of Snakebite Threat

The perceived fatality rate of untreated bites from dangerous snakes reported in surveys (Table 3) was significantly higher among participants who had not attended workshops (mean of 89%, whether or not they had spoken to an attendee) compared to those who did attend workshops (mean of 33%) (comparing attendees with non-attendees who had not spoken to attendees, binomial regression coefficient = 2.826, SE = 0.650, z = 4.346, *p* = 1.39 × 10^−5^; comparing attendees with non-attendees who had spoken to attendees, binomial regression coefficient = 2.830, SE = 1.218, z = 2.323, *p* = 0.020).

### 2.4. Practice

#### 2.4.1. Personal Treatment of Snakebite

**Figure 3:**
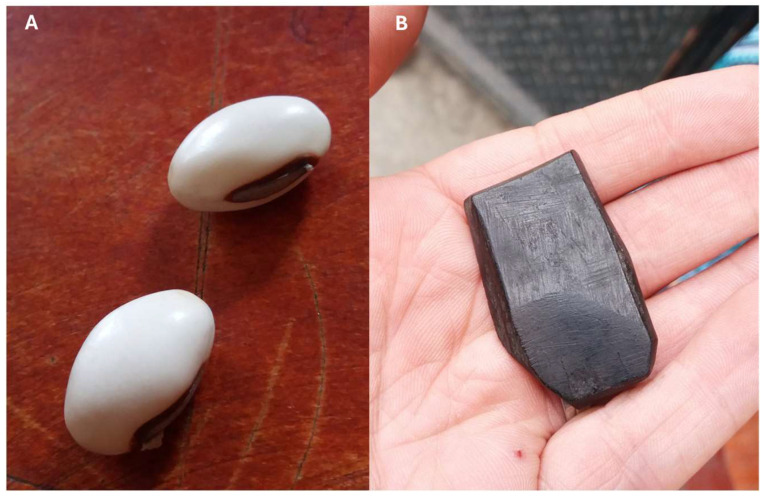
Common traditional methods of snakebite treatment in this area (**A**) snake beans, likely to be *Canavalia ensiformis*, as reported from the Rwenzori region of Western Uganda [41] and (**B**) a black stone, which is a community-held thin, smooth, and black stone.

When asked how they would react if they themselves were bitten by a snake that they believed was dangerous (Question 7), survey respondents usually gave between one and four responses (Table 5). Amongst the 43 non-attendees who had not heard of the workshops, the most commonly listed response was to seek hospital treatment (56%). The second most frequent response from non-attendees was to kill the snake (49% suggested this, with 37% suggesting this as their first response) (Table 5). Traditional healing approaches were also frequently listed, including drinking old ladies’ urine, tying a cloth around and/or cutting the wound, and applying snake beans and black stones to the wound (Figure 3).

Data from interviewees, when asked what they would do if they were dealing with a snakebite incident, revealed a similar pattern, but with fewer treatments mentioned (Table S4). Amongst non-attendees, over half of the participants said they would seek hospital treatment, but typically after using unspecified ‘local medicine’, tying tourniquets to the victim, or using the local ‘snake beans’ (Figure 3A). One workshop attendee (interviewee 6) also stated that those in the community who have not been to (or heard about) the workshop still cut the bite site and use snake beans.

#### 2.4.2. Association of Workshop with Personal Treatment of Snakebite

For the eight survey respondents who had not attended the workshop but had spoken to someone who had, the responses were similar to those who had not attended nor spoken to anyone who had, with the three most popular suggestions being to seek hospital treatment, kill the snake, and drink old ladies’ urine (Table 5). However, for those who attended the workshop, responses were substantially different, with most participants (76%) suggesting going to hospital as the primary or only response. Some more traditional treatments were also suggested by a minority (≤12%) of workshop attendees (Table 5). In contrast to non-attendees of both groups, no workshop attendees suggested to kill the snake as a response to snakebites. Attendees also demonstrated greater knowledge regarding local snake handlers and local hospital transport than non-attendees did, with 17 of the 19 respondents who suggested hospital treatment also stating that they would call a local free motorbike transport service to the hospital.

Similar patterns were seen in interview data (Table S4). Of the 10 interviewees who had attended the workshop, all of them stated they would seek hospital treatment, either as the sole response or combined only with reassuring the victim to keep them calm. In comparison, fewer non-attendees suggested hospital and more suggested tying cloths, using snake beans or other traditional medicine.

#### 2.4.3. Community Norms in Snakebite Treatment

Question 6 of the survey asked what treatments have been given to people who have been bitten in the local area. The most common survey response from participants who had not attended workshops was to drink old ladies’ urine (56%), followed by using snake beans (51%), black stones (47%), and tying a cloth or similar item around the wound (47%) (Table S5). Less frequently suggested treatments involved drinking palm oil (28%), remaining still (19%), and cutting around the wound (16%) (Table S5). Faeces were suggested by one participant, although they did not state how this would be used in snakebite treatment (Table S5).

#### 2.4.4. Association of Workshop with Suggested Community Norms for Bite Treatment

The eight survey respondents who had not attended the workshops but had spoken to an attendee gave similar responses to question 6 as non-attendees (Table S5). Of the workshop attendees, the most frequently mentioned treatment was going to hospital (72%) which was in strong contrast to non-attendees, none of whom suggested going to hospital. Traditional methods were mentioned by attendees alongside going to hospital (Table S5). Killing the snake was not present within any responses to survey question 6, although tying the wound with the head of the snake was mentioned by one workshop attendee.

#### 2.4.5. Snakebite Prevention in the Community

Interviewees were asked whether there is anything they do to avoid snakes or prevent snakebites, and then also if they know of anything others do to prevent snakebite. Non-attendees reported several practices which are at least partly intended to avoid snakebites, with an average of ~2 methods per individual non-attendee (Table 6), though two non-attendees who had spoken to someone from the workshops were unaware of anything they could do to reduce encounters with snakes or snakebites.

When interviewees were asked what prevention strategies other people used, 10 methods were brought up, and these overlapped heavily with the personally reported ones (Table S6). In addition to those suggested above, two non-attendees (who had not spoken with an attendee) reported the use of paraffin spread around as a repellent, and one reported that urinating in a bucket and leaving this uncovered in the bedroom was practiced where they grew up, near the forest. The latter was believed to entice the snakes to drink from the bucket and that this would make them “lose the morale of biting somebody”. This question also received responses related to practices once a snake is encountered, with killing the snake being the most common of these, and one attendee reporting that they have told others they can leave the snakes alone and that some people they know have taken this on board.

#### 2.4.6. Association of Workshop with Snakebite Prevention

During interviews, workshop attendees reported an average of three methods each to reduce snake encounters or bites (Table 6) compared to two for non-attendees, and so appeared to be more aware of strategies to prevent snakebites. All attendees were aware of strategies to prevent snakebites, and all methods reported are likely to be effective. Of the 10 interviewees who attended the workshop, all stated that they have changed their practices to improve snakebite prevention as a result of the workshop. The methods now used by workshop attendees to prevent snakebite heavily overlapped with those identified by the communities during the workshops (Table S7).

#### 2.4.7. Encounters with Snakes and Actions

Question 2 from our interviews asked how often the participants see snakes around their community, and question 10 asked what they would do if they came across a snake. Encounters with snakes are frequent in this area, with 18 of the 21 interviewees reporting that they typically encounter snakes one to four times a month, with those who mentioned variation stating that they find snakes more often in the rainy season and on sunny days. The remaining three reported lower encounter rates, from <1 per month to <1 per year.

Two workshop attendees specifically reported that they have seen fewer snakes since implementing prevention measures that they learned from attending:

*“The rate at which we used to see them before the workshop has reduced because I have slashed all the compound, but I used to see almost two in two months and now they have reduced and have not seen some after cleaning my compound. In a month I would see like three/two when we had not learnt how to keep them off.”*

(Interviewee 11, Attendee)

*“I used to see many snakes before the workshop in my house but now I think we have prevented them I no longer see them around in our community…”*

(Interviewee 19, Attendee)

Snakes were typically encountered around the home, sometimes inside but mostly in the garden/compound area and usually in the bushes or trees, or when digging or cutting plants such as plantains, which are commonly grown at home. Snakes were typically observed basking or fleeing, with common mention of a ‘coiled’ posture; only one interviewee used the word ‘aggressive’, which was then clarified to mean the snake fled quickly. Of the 21 interviewees, all stated this was the most common scenario when they come across snakes, with one participant also stating they find them regularly under stones that are moved for building, one reporting seeing snakes crossing the road when the person is walking around, and two stating that they also see snakes drinking or swimming at a lake.

**Figure 4:**
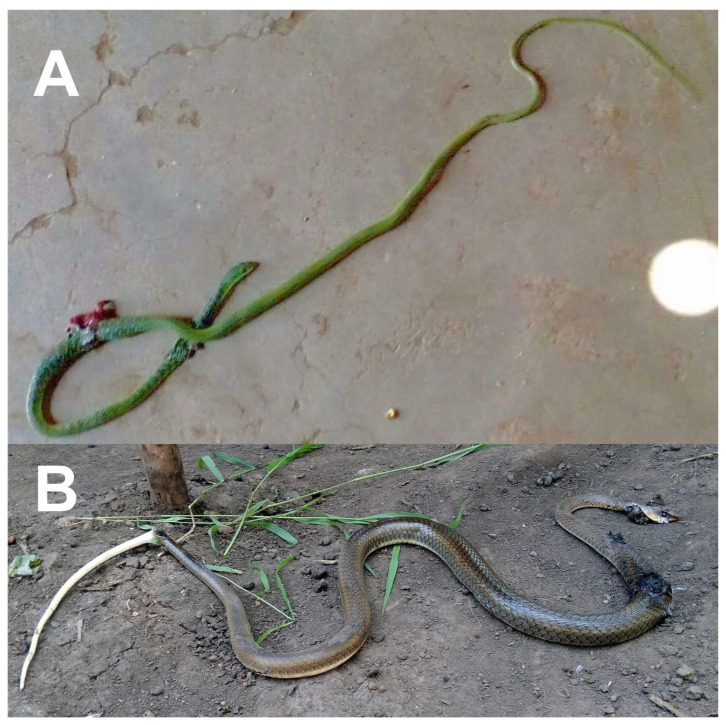
Snakes killed by anonymous members of the community. (**A**) A green bush snake, *Philothamnus* sp., and (**B**) an olive sand snake, *Psammophis mossambicus*. Neither of these snakes are dangerous, either being completely harmless (**A**) or possessing a very clinically mild venom, posing no significant threat to humans (**B**). Note that photographs were taken by researchers opportunistically; we did not request nor encourage community members to provide or photograph dead snakes and hence provided no motivation to kill snakes.

Reported actions of people when they come across a snake comprised only two general options: killing the snake (Figure 4) or stepping back and leaving it alone (Table 7). Workshop attendance was associated with a greater likelihood of letting the snake go on its own rather than killing it (Fisher’s Exact Test: *p* = 0.005; Table 7). Of the 10 attendees, all said they have changed their response to snakes since the workshops, with all 6 who elaborated stating explicitly that they used to kill the snakes. Interviewee 10 (attendee) said they would give it space and let it go away on its own, but specified that if it was in the house, they would call trained snake removers to remove it. Moreover, of non-attendees who had spoken to an attendee about the workshop, three of them said they used to kill the snake but that since speaking to attendees, they would now just leave the snake to go on its way.

#### 2.4.8. Key Learning from the Workshop

For workshop attendees, we asked two questions during the interviews to understand what key messages they have taken away from the workshops ~10 months later. The first of these simply asked ‘What did you learn from the workshop’, and the most consistent response (9 out of 10 interviewed attendees) was about how they learned that they can ‘live with snakes in harmony’ and do not have to kill them. The one respondent who did not say that instead highlighted that they learned about the importance of snakes; another learning related to a more positive attitude. Methods for preventing encounters with snakes or snakebites were also frequent, with eight participants stating this as something they learned. Three participants also mentioned learning about the importance of snakes to the people and/or natural environment. One participant specifically mentioned the WHO video shown during the workshop as teaching them not to use traditional treatment methods and instead go straight to hospital.

When asked specifically if they could ‘remember any of the things we discussed to prevent snakebites in the workshop’, notwithstanding those strategies mentioned earlier, all participants were able to relay two to five methods. These reflected the methods given in Table 6 and Table S7, with wearing closed shoes and maintaining the house and garden to reduce hiding places being the most commonly reported (6 and 5 of 10 attendees who were interviewed, respectively).

#### 2.4.9. Dissemination of Information from the Workshop

Interviewees who had attended the workshop, and those who had heard about it from someone who had, were asked who they had spoken to and what they were told. All attendees reported that they had spoken to others in their community about the workshop: mostly family, friends, and neighbours in the community. Three of the ten interviewed attendees also reported speaking to women’s groups in the community, which they are members of. One respondent is a member of the Village Health Team (VHT) and spoke to other VHT members as well as spreading information from the workshop to other community members in their VHT role.

The most common information reported to be disseminated was that is better and safer to leave snakes alone to go their own way (8 of 10 respondents). More generally, participants reported talking to people about how they can live with snakes (7 of 10 respondents), how to prevent snakebites (5 of 10 respondents), and the benefits and importance of snakes to people and nature (3 of 10 respondents). One participant reported telling people that if a bite happens, they should go to hospital rather than rely on traditional treatments, and that snakes are often encountered when the snakes are looking for food, rather than seeking to bite humans.

Non-attendees who had spoken to an attendee generally confirmed these dissemination patterns, with four of seven respondents stating that they had heard it from friends or family, and the other three having heard from other community members. The most common messages they recalled were about how to prevent snakebites (five respondents) and how to ‘live in harmony’ with snakes (four respondents). One respondent also reported being told of the importance of snakes, and another that “sometimes snake are good, sometimes they are very bad”.

## 3. Discussion

### 3.1. Summary

This exploratory study demonstrates the wealth of contextually specific knowledge, attitudes, and practices (KAP) around snakes and snakebites held by a rural Ugandan community. We found that community members who had not attended the workshop had a working knowledge of some local snake species, but found them difficult to identify from images (Table S1). With the exception of the brown house snake (a common and harmless snake), all other species were generally considered to be dangerous, regardless of the risk they pose (Table S3). Attitudes towards snakes were generally negative, with community members reporting fear of venomous bites as driving this negative perception (Table 1). This likely relates to the perceived high mortality risk from bites, with survey participants who had not attended workshops estimating an 89% mortality risk from untreated bites from dangerous snakes (Table 3). However, many people were also able to identify the potential benefits of snakes, including use of their leather, vermin control, tourism, and potential medicinal uses (Table 2). The most frequent suggestions for treatment if they themselves were bitten included going to hospital and killing the snake (Table 5). Traditional treatment methods were also commonly suggested, including using black stones, snake beans, drinking old ladies’ urine, and tying cloth or other items around the wound (Figure 3, Table 5, Tables S4 and S5). Non-attendees were able to suggest several snakebite prevention methods, with the most common involving keeping the house and surrounding area well maintained (Table 6).

We found substantial differences in KAP between those who had attended our participatory workshops and those who had not, and these differences are still apparent 10 months after the workshops (when interviews were conducted). Workshop attendees were better at identifying snake species from photographs (Figure 1, Table S1), but were not better at assessing the risk posed by these species, and were more likely to incorrectly consider a snake to be harmless than non-attendees (Figure 2, Table S3). Attitudes towards snakes were generally more positive in workshop attendees, although fear of snakes was still frequently reported (Table 1). We found large differences in suggested snakebite treatment practices, with 76% of survey respondents and 100% interviewees who had attended workshops suggesting hospital treatment for snakebite, usually as their primary or only response (Table 5). Notably, no workshop attendees suggested killing the snake as a response to snakebite, in contrast to nearly half of the non-attendees (Table 5). In terms of snakebite prevention, attendees were able to suggest a wider variety of prevention methods than non-attendees and indicated that they had implemented these methods since attending the workshop (Table 6). Finally, workshop attendees did not suggest killing snakes as a response to coming across a snake and instead suggested giving it space and letting it go, unlike non-attendees who frequently suggested killing the snake (Table 7), which could potentially lead to bites (Table 4). While we could not robustly compare KAP pre- and post-intervention, due to the impact of COVID-19, our study suggests that community engagement workshops can increase knowledge of snakes, boost positive perceptions of snakes, and drive changes in practices relating to snakebite treatment and prevention in the community. However, future modifications of the workshop may be necessary to ensure that risks posed by snakes are not underestimated. Below, we discuss our results in detail and put them into a broader context.

### 3.2. Knowledge of Local Snake Species

Our survey and interviews demonstrated that community members have a working knowledge of some of the commonly found snakes in their local area, with five species being frequently identified and described during interviews (Table S2). However, there are 48 species of snake that are likely present in the area, so participants only described a relatively small proportion of these, potentially those that are particularly common, distinctive and/or dangerous [42,43]. Community members who had not attended workshops were usually unable to identify snake species from the photographs we showed them (with the exception of the brown house snake and brown forest cobra in a hooded position, Table S1). This is in keeping with studies in the USA and Australia, which have also shown a limited ability to identify local snake species [44,45]. Interestingly, in our survey, the photograph of a forest cobra without its distinctive hood proved difficult for participants to identify in comparison to a picture of the same species with its hood, suggesting that humans recognise the distinctive defensive posture as an aposematic warning signal [46,47]. Furthermore, some interview participants described lizards and crocodiles when asked about snakes, likely because *enjoka*, the local word for snake, can also be interpreted in some dialects to mean scaled animal more generally. Thus, clarification of language may be needed in future iterations of the project. Such complexities emphasise the need for more detailed understanding of community level snakebite knowledge, which could be most effectively achieved by qualitative studies similar to that of Duda et al. [48], who explored lay-knowledge of snakes within central Africa via anthropological and ethnohistoric approaches.

Our workshops did not focus on teaching snake species identification. This was because we were concerned that the workshops were of an insufficient duration to leave participants capable of correctly identifying dangerous snakes in the medium to long term [49], potentially leading to an increase in bites due to misidentification. Instead, we showed participants a poster with photographs of different local snake species, with the level of harmfulness to humans indicated using a traffic-light system. This was primarily to highlight the point that not all snakes are harmful (only a quarter of local species are dangerous to humans). The poster was also used to draw attention to the difficulties in easily distinguishing between dangerous and harmless species, and so we deliberately avoided encouraging participants to try to identify snakes they find. Our finding that workshop attendees were able to identify more snake species from photographs than non-attendees (Figure 1) was therefore surprising. This difference in snake identification could potentially be explained by participants’ interest in snakes being spurred on by their attendance at the workshops. Alternatively, the selection process for workshop participation may have resulted in those with a prior interest in snakes preferentially attending [50]. However, the widespread negative attitudes towards snakes at the start of workshops suggests that participants were not unusually interested in the snakes themselves. Finally, this finding may be influenced by a reduction in fear of snakes in workshop attendees, as in the USA, those people with the greatest fear response to snakes also had the most challenges in identifying species [51]. In our current study, it was not possible to conduct surveys and interviews of the same participants before and after workshops due to the impact of COVID-19, but future work using this approach may be able to differentiate between these possibilities.

### 3.3. Risk Assessment of Local Snake Species

In the present study, participants who had not attended the workshop considered almost all snake species (with the exception of the brown house snake) to be dangerous (Table S3). However, most workshop attendees were aware that many species are harmless to humans, a point covered during the workshops. Interestingly, this appears to have led to workshop attendees being more likely to misidentify a snake as harmless in photographs compared to non-attendees, while the opposite (and potentially safer) position characterised the errors of non-attendees, which tended to consider almost all harmless snakes to be dangerous (Figure 2). This could cause problems if our workshop content resulted in an increase in unsafe behaviours around dangerous snakes. However, our interview data suggest that this is unlikely, as workshop attendees reported more appropriate practices in relation to snakebite prevention and treatment compared to non-attendees. An alternative explanation for this result is that participants now consider snakes harmless *if left alone*, and it is important to note that even if they believe that a snake is likely to be safe, that does not mean they will interact with it. Nevertheless, such risks should be carefully considered in future versions of our workshops and within similar interventions in other contexts.

### 3.4. Attitudes Towards Snakes

In line with other studies in areas containing snakes that pose danger to people [42,51,52,53,54], community members had a generally negative attitude towards snakes, reporting high levels of fear (Table 1). However, workshop attendees often demonstrated a willingness to tolerate snakes, reduced aggression toward snakes, and a less fearful attitude toward snakes, and frequently cited the workshop content relating to snake behaviour as being a driving force in their change in attitude. During workshops, we attempted to generate empathy towards snakes by requesting that the participants imagine that they were snakes and suggesting how they would behave in different situations: for example, when too hot or cold, or when threatened or attacked. Empathy has been identified as a strong predictor of tolerance towards ‘problem species’, and may be more important than monetary benefits in promoting human–wildlife coexistence [55].

Although attitudes towards snakes were often fearful, both workshop attendees and the wider community could describe some benefits of snakes, such as killing rodents and attracting tourists (Table 2). This acknowledgement of snake value is important, as it provides a pathway for which negative attitudes and practices relating to snakes can be challenged within the context of local knowledge [56]. Discussing positive qualities can allow people to shift negative attitudes and thereby influence practices such as snake-killing behaviour. However, one does not want to encourage unnecessary contact with snakes, even if this is driven by positive curiosity. The participatory nature of workshops is well-suited to exploring such nuance, as participants can ask questions and the facilitator can check if any messages need clarifying or correcting.

### 3.5. Perception of Snakebite Threat

Almost all participants in this study could recall a snakebite experience within their community, with survey respondents recalling a total of 178 bites and all but one interviewee being aware of at least one bite recently (Table 3). Six of these bites were believed to have resulted in death, which is consistent with the estimates of case fatality rates from global numbers of envenomations and deaths from snakebite [1]. However, there does appear to be a disconnect between experiences of snakebite fatalities and attitudes toward the risk of snakebite. The survey data shows that non-workshop attendees believed untreated snakebites to be fatal in most cases, when only a handful of fatal bites were reported by these same people. This mismatch is central to snakebite prevention, as fear of snakebite may drive delays in care, stigma, and snake killing [52,57]. Expectations of mortality may also partially explain the reliance on traditional treatments, because any incident of bite survival following traditional treatment will reinforce perceptions of the treatment’s efficacy, even if there was no envenomation: for instance, from dry bites or those from a non-venomous snake. Indeed, this point was spontaneously made by a workshop participant upon learning that not all snakes are venomous, and that even venomous species can give dry bites. Interestingly, workshop attendees mostly suggested high mortality rates when they themselves had experienced snakebite fatalities in their communities. This result not only demonstrates the impact of personal experience upon attitudes but also provides a ‘hook’ with which to frame discussions around snake risks [48,56,58,59,60]. However, one needs to strike a balance between reflecting on lived experiences and maintaining a healthy respect for snakebite potential. Promisingly, the discussion of different snake species, their health risks, and behaviours does appear to have provided workshop participants with a more nuanced understanding.

### 3.6. Practices in Snakebite Treatment and Prevention

Practices relating to snakebites treatment were varied and included a range of traditional options such as black stones and snake beans (Figure 3), cutting the wound, topical application of herb mixtures, applying a tourniquet, and drinking an old woman’s urine, alongside visiting the hospital (Table 5, Tables S4 and S5). Many of these traditional methods are widespread on a global scale, with black stones being disproportionately common in Africa [61,62]; however, others appear to be much more local. For instance, the snake bean we report here has been recorded as a snakebite treatment in the immediately adjacent Rwenzori region in Western Uganda [41], but not in Northern Uganda [63]. Similarly, drinking the urine of an old woman was very commonly reported in our study, but we can find no record of this as a treatment for snakebite in the literature, despite reports of drinking the victim’s own urine in other parts of Africa [64,65,66]. Going to hospital was also suggested by both workshop attendees and non-attendees, but there was a notable increase in suggesting hospital treatment for attendees, with this response often being the first and only response suggested when asked how they would respond if they themselves were bitten. These findings demonstrate the value of locally based, participatory approach to explore traditional practices in relation to modern medicine. At no point did facilitators directly contradict traditional practices, as that would risk damaging the trust we have built with the communities, but we showed the WHO snakebite prevention and first aid video [67] as part of the workshop. This allowed us to convey additional key points and more sensitive advice from a slightly more detached and indirect perspective, while more directly using the video to emphasise the success of the community in devising good snakebite prevention interventions from their own knowledge, and hence we continued to build and maintain trustworthiness.

Both snakes and the communities that live amongst them are likely to benefit from wide use of safe and ecologically sound snakebites prevention measures. Our workshops found that communities already have a considerable knowledge of prevention measures, primarily involving maintaining the areas inside and around buildings to avoid the build-up of materials that snakes may seek refuge in (Table S7). The workshops appeared to aid in the discussion and uptake of prevention methods, with 8 of 10 interviewees who had attended workshops stating that they learned new prevention methods during the workshops (listed in Table 6). These measures aligned well with the circumstances of bites described by the community during interviews, with 20% occurring in the home, 33% occurring while performing manual work outside, 33% whilst walking in the dark, and 7% while attempting to kill a snake (Table 4). The prevention methods identified and put into practice by community members therefore have substantial potential to reduce snakebite.

Although there was little evidence that speaking to an attendee improves knowledge or attitudes towards snakes, there is more of an indication that it can influence practices related to snakes when encountered, or ways to prevent snakebites. This was not only more closely tied to the main aims of the workshops, but also corresponds with the key information reported by attendees to be conveyed to others and reported by those they had spoken to as being received. However, it is clear that some of the many elements of the workshop are not yet disseminating widely across this community. This suggests the workshop may need a more explicit activity around disseminating learnings to the wider community.

### 3.7. Snake Killing

Our study found that snake killing is a common response to both finding a snake (Figure 4, Table 7) and to snakebites (Table 5). For example, almost half of the survey participants who did not attend the workshop suggested that they would kill the snake in response to a bite. In contrast, this response was not suggested by any workshop attendees. This could be due to an increase in knowledge of snakebite risks generated during the workshop. Demonstrating the risk associated with attempting to kill snakes was a key component of the workshops and a specific role-play activity was included to show just how close to a snake a human must become to kill it and, in turn, how easy it is to receive a bite from this proximity. Our interviews also confirmed that this risk was sometimes realised, with interviewees reporting bites occurring whilst the victim was actively trying to kill a snake (Table 4). The need to minimise human–snake conflict to minimise bite risks has also been noted in the wider literature [59]. The community’s perception of most snakes as dangerous only reinforces the need to keep our messaging and activity focused on avoiding approaching or killing snakes within the workshops.

In addition to reducing snakebites risk, promoting human–snake coexistence may also reduce snake persecution and assist with their conservation. As well as being the second most common threat to snakes globally (behind only habitat loss), ‘persecution or control’ is also the single most common threat to Ugandan snakes [9]. Rather than trying to reconcile the danger posed by certain snake species, this research team believe it is imperative to explore alternatives to harming and killing snakes. Very few studies globally focus on minimising snakebite, or broader human–snake interactions, to protect snakes [56], yet human–snake conflict is a biodiversity issue and is essential to address in terms of promoting human–snake coexistence [52]. Several reviews of human–wildlife conflict and coexistence have emphasised the need to work alongside stakeholders in the affected communities to understand perceptions underlying the conflict and generate alternative solutions [68,69,70]. These reviews also emphasise that human–wildlife conflict is a dual issue of impacts on humans and conservation of the animal species, yet this perspective remains most widely incorporated in discussions of large and charismatic mammals, such as big cats and elephants, rather than in reptiles such as snakes [68,69].

### 3.8. Limitations and Future Research

This was a small-scale study conducted in a specific rural area of southwestern Uganda and thus, caution should be taken in relation to generalising findings beyond the specific study context, particularly as local contexts of snakebites vary widely. Furthermore, due to COVID-19, we were unable to conduct pre-intervention interviews and surveys, and instead focused on comparing workshop attendees and non-attendees. Future interventions should involve both pre- and post-intervention assessments to better gauge changes in KAP in relation to workshops. In addition, due the pilot nature of the study, surveys were distributed based on availability and accessibility, with some targeted sampling to obtain a sufficient sample of individuals who had attended workshops (approximately half of our survey participants attended the workshops). This may introduce biases to our dataset if the KAP of our respondents does not represent the population, and future studies should both collect data on the baseline characteristics of the participants and assess whether these are likely to represent the population as a whole. Nevertheless, we find evidence that those who had attended snakebite prevention workshops had differing KAP regarding snakes and snakebite than those who did not attend. Furthermore, attendees often cited the workshop as a driver of change during interviews, suggesting that workshops had a lasting impact on attendee KAP, with key messages being retained by attendees for at least 10 months.

Whilst we found evidence that workshops may impact KAP, we were limited to asking about self-reported changes in attitudes and practice. Although participants had clearly understood the meaning and purpose of the workshops (for example, they were aware of more potential benefits of snakes and a greater variety of snakebites prevention methods), a desire to please the interviewers may have led to an over-reporting of changes in attitudes and practices. To overcome this, future studies could use interviewers that were not involved in the workshops and also incorporate more objective measures of changes in practice. Ideally, researchers would collect data on the number and severity of snakebites in the community, along with the number of snake killings and the species concerned. However, this data can be very challenging to collect. Some studies have assessed changes in practice through monitoring the number of hospital admissions for snakebite (e.g., [42]); however, these data are often difficult to interpret because hospital admissions are impacted by both the number of bites occurring (which researchers aim to decrease post-workshop) and also the desire and ability of bite victims to attend hospital (which researchers aim to increase post-workshop). On a local level, Village Health Teams may be able to collect information on the number of bites and the treatment of snakebites in the communities, but this would require careful evaluation before implementation.

Our study purely focused on the KAP of community members and therefore did not assess or map the snakebites landscape in terms of surveying snake diversity and prevalence, nor the existence, location, and quality of snakebite treatment centres and their resources. This is an essential next step within the project, as improved local knowledge of snake species and their prevalence will allow workshops to become more specific and consider the reality of snakebite risk in the area. This is perhaps especially pertinent for Uganda, where basic knowledge of snake species and their distribution are limited [19]: particularly the more poorly surveyed western regions. Equally, a clear understanding of what treatment options are available is essential if our key message around treatment is to remain in line with WHO guidance [67,71]: “Go to hospital or seek the advice of a trained health professional”. Unfortunately, many health centres and even large hospitals in Uganda are ill-equipped to deal with snakebite due to a lack of appropriate medication, equipment, and training [22,23]. Addressing these systemic aspects of the snakebite challenge is also essential to build trust in communities that there are appropriate alternatives to traditional medicine.

The study revealed some methodological challenges around applying community engagement approaches to snakebite prevention. For example, whilst interviewees could name local snakes when asked open questions, the survey participants were not as readily able to identify some of the same species of snake from photographs. There could be many reasons for this, including the fact that photographs may have been taken in other countries and may display differences in the size and colours of snakes to those that this community usually interact with. Eyesight issues, colour blindness, and poor lighting in the workshops could also affect participants’ ability to engage with photographs. Thus, in future interventions, any photographic prompts should be pre-tested to ensure participants can clearly see and interact with them within the physical spaces in which the research is taking place. Alternatively, open questions such as ‘does enyarubabi look like any of these pictures; if so, which ones?’ rather than ‘which one of these pictures is enyarubabi?’ may put less pressure on participants and bring the focus back to knowledge exchange, rather than getting an answer right or wrong (which may have led participants to be nervous about committing to an answer).

## 4. Conclusions

This exploratory study piloted a participatory workshop format aimed at promoting human–snake coexistence and collected both qualitative and quantitative data from attendees and non-attendees after workshop delivery. The interaction between the COVID-19 pandemic and funding deadlines impeded our ability to collect pre-intervention Knowledge Attitude and Practice (KAP) data, which would be required to robustly evaluate workshops. However, our post-workshop interview and surveys, plus workshop reports, yield important learnings which will support the refinement of future content but also identify pathways-to-impact. Firstly, workshops were well-attended despite a lack of financial incentive, suggesting that the topic was of interest to this community. Indeed, all datasets suggest a working knowledge of snake species in the area, and a variety of common strategies used to react to snakes and treat snakebites. Such contextual knowledge is essential to shaping future interventions and seeking alternatives to potentially harmful behaviours. Secondly, all data suggests a common fear of snakes within this community, yet participants also describe the benefits of snakes: killing rodents, attracting tourists, and being used in traditional crafts. The concept of snake benefits could be used as a ‘hook’ to challenge fearful beliefs and human–snake conflict in future interventions. Thirdly, although workshop attendees described aggression as an initial reaction to snakes in the workshops, in later interview and survey responses, they primarily suggested less aggressive responses, such as avoidance, and cited the workshop as a driver of this change. It thus appears that messages of human–snake coexistence were retained. Finally, workshop participants displayed knowledge of potential snakebite prevention methods and the importance of seeking hospital treatment after a bite, again citing the workshop as a driver of change. Taken together, these findings suggest that participatory workshops focused on promoting human–snake coexistence could be an effective intervention to mitigate snakebite. Delivery at scale and evaluation via a robust framework collecting both pre- and post-intervention data is now required to fully determine the impact of such workshops on long-term snakebite prevention outcomes.

## 5. Materials and Methods

### 5.1. Field Site and Study Population

This study took place in the Rubirizi district, southwestern Uganda, which is located approximately 364 km from the capital Kampala. The 2020 mid-year census results estimate the population size to be around 144,100 people with an annual growth rate of 1.9% between 2014 and 2020. Settlements across the region are predominantly rural, with most households owning land, and subsistence farming accounted for 78.8% of the region’s income in the 2014 census. However, over 50% of Queen Elizabeth National Park is located within the Rubirizi region, meaning that many households also gain income from tourism or direct National Park employment as rangers, cooks, cleaners, administrative staff, etc., albeit on a seasonal basis. Although transport to hospital is often not available as standard in this area, the project team have set up a community motorbike ambulance, which can be used (with no charge) to access medical care for snakebites and other conditions by the communities in the area.

### 5.2. Study Design

We first delivered a series of half-day participatory workshops, designed by the project team (Table 8). Workshops aimed to promote human–snake coexistence whilst also exchanging knowledge on snake species, behaviour, and biology with community members. Second, a mixed method survey was conducted to investigate the KAP of community members in relation to workshop attendance. Finally, we conducted interviews of community members in relation to workshop attendance, allowing us to gain more detailed information about their learning from workshops, and whether this had influenced their KAP. Survey and interview instruments are available in the Supplementary Materials.

#### 5.2.1. Workshops

Half-day participatory workshops were designed by the project team (Table 8). Workshops aimed to promote human–snake coexistence whilst also exchanging knowledge about snake species, behaviour, and biology with community members. Workshops followed a participatory format, including plenary sessions, visual and outdoor activities, question and answer sessions, and a viewing of the World Health Organisation snakebite video (see below for further details).

Participants were recruited to workshops via word of mouth, spread by community leaders in the local school and the Village Health Team (the basic unit of primary care in Uganda). Participants were asked to express an interest in joining the workshop and were then assigned a day to attend. Consent was taken in the first hour of the workshop, with participants being read the information sheet, given time to ask questions over breakfast, and leave if they wished. Participants wishing to continue with the workshop signed a consent form and also provided separate consent for photographs to be taken, which did not impact their participation in the project.

Seven workshops were delivered by the project team in July 2022 and May 2023. Workshops were delivered in English by team members 3 and 4 (with some assistance from team members 2 and 5 and occasionally other volunteers). Translations into the local language (Runyakitara) were provided by team member 3. Team members 1 and 5 cross-checked understanding and often provided English-to-English translations, using the local dialect. Team member 6 completed an observation for three of the July 2022 workshops to capture the knowledge exchanged between the community and research team, the community’s observed reactions and engagement with each activity, and the feasibility of delivery, including areas within the workshop which required more detailed clarification or explanation.

#### 5.2.2. Workshop Structure

Workshops lasted for approximately 4 h and consisted of seven sections.

(1) We first conveyed basic knowledge of snake biology which might result in snake-human conflict. This comprised an introductory talk on basic aspects of snake behaviour that are relevant to reducing snakebites, including thermoregulation (seeking sun and shade) and seeking refuge in ‘safe’ places. We then tried to develop empathy with the snake through engaging the participants in a thought experiment. In this activity, participants were asked to imagine that they were a snake which has entered a home while trying to find a safe and cool place and considering what they would do if threatened by an aggressive human. This aims to help understand why snakes bite or may appear aggressive, and why they sometimes turn up in the same place as humans (i.e., both seeking the same things, rather than the snakes looking to find and bite humans).

(2) We moved on to consider the potential benefits of having snakes in the environment through asking participants if they could think of any benefits that snakes might bring to them.

(3) We then explored some risks of snakebites in the local environment. This started with an acted-out situation to demonstrate the dangers of approaching snakes to kill them. Here, one person acted out the snake role (with their hand representing the snake’s head) and another person acted as a person trying to kill the snake with a stick. The snake could only successfully bite when the person got close enough. We then briefly explored local snake species, first asking how many types of snakes the participants think there are in their area, before asking how many are dangerous. We then gave the correct answer to these questions and showed a poster with all local snakes, colour-coded by risk, which helped to emphasise that, although some snakes are very dangerous to people, most snakes are not dangerous and bring lots of benefits, as the participants identified earlier. However, we also used the poster to emphasise that some dangerous snakes look very similar to harmless snakes, so appropriate caution should always be taken.

(4) We then moved on to co-develop snakebite prevention methods in the local area. This began with a walk around the local area, where participants used their past experiences of snakes, along with the information covered earlier on snake biology, to identify places where they thought snakes might want to be. When they identified a location, we asked whether there was anything that could be done to prevent snakes being around that area or coming into contact with people. Although this usually resulted in key solutions (or locally feasible alternatives) being identified by the participants, we sometimes also used prompts and helped to guide the activity where necessary.

(5) After a break, we put the participants into groups of ~5 to discuss what they currently do to prevent snakebites, and what they could do in addition to that, based on the previous content/discussions or new ideas, focussed strongly on those that are acceptable, appropriate, and feasible for their communities.

(6) We briefly covered snakebite treatment through showing participants a short WHO video on snakebite prevention and first aid [67]. We chose to do this, rather than give advice ourselves, firstly because none of the delivery team are medically qualified (which has risks of responsibility and of damaging trust), and secondly to bring up issues such as the use of some traditional approaches without potentially developing conflict between ourselves and community members. After the video, we highlighted that the snakebite prevention ideas and suggestions the participants have come up with themselves align very well with the WHO guidelines. This indirect approach to what to do in the event of a bite enabled the team to convey key points clearly while focusing more directly on the community-led solutions.

(7) Finally, we ended the workshops with the opportunity to ask any final questions about the workshop material, and we requested feedback via asking how the workshops could be ‘even better if…’. We also thanked all participants for their time, engagement, and help with co-developing snakebite prevention solutions.

#### 5.2.3. Survey and Interview Procedures

##### Survey

A mixed method survey (see Supplementary Materials) was conducted between July 2022 and August 2023 by team member 3, who is fluent in English, the local community dialect of English, and the local language (Runyakitara). Surveys were provided to participants recruited by team member 3 by direct solicitation, assisted by Village Health Teams. A combination of convenience and purposive sampling was used. This ensured that respondents comprised some individuals who had attended the workshops and others that had not attended the workshops (and who may or may not have spoken to those who did). A consent form was provided to all participants before they engaged in any research activities for the project, which was available in both English and Runyakitara, and with team member 3 reading the form aloud where necessary (e.g., if participants were unable to read).

##### Interviews

A semi-structured interview guide was developed by all team members (see Supplementary Materials) and focused on questions relating to knowledge, attitudes, and practices around snakes, snakebites, and snakebite treatment. Interviewees were recruited by word-of-mouth by team member 3, who is embedded within this local community and via his connections to local community gate keepers and village health teams. Recruited participants provided verbal consent, which was captured in the interview recording and subsequent transcript, in addition to the consent forms mentioned above. We refrain from publishing the individual-level demographic data we collected (age, education level, and gender), because in this community, even this level of detail may lead to identifiable individuals, particularly for more highly educated people. Our ethical approvals and participants’ consent do not allow us to publish information which may be personally identifiable. However, in summary, our interviewees ranged within ages 18–50, were educated to levels from Primary 3 to Higher National Diploma, and had a slightly female-biassed gender ratio (15 females and 6 males). We examined the individual data and considered that this is broadly representative of the wider community, based on the 2024 Census data (https://statistics.ubos.org/nphc/drilldown?subregion=41&district=425, accessed on 18 December 2025); notably, the region is also female-biassed, such that our data are not different from the 57% female representation (exact binomial test: sample proportion = 0.71, expected proportion = 0.57, *p* = 0.27). Interviews were conducted between 7th and 9th May 2023 by team members 3 and 4, both of whom are fluent in English, and team member 3 is also fluent in the local community dialect of English and the local languages. Interviews were recorded on a Digital Voice Recorder (Evistr L157 16gb) and then typed up verbatim into English for analysis. Similarly to surveys, a mixture of workshop attendees and non-attendees were interviewed, and (for attendees) interviews were conducted approximately 10 months following attendance at a workshop; those who attended workshops in July 2022 were interviewed in May 2023.

### 5.3. Statistical and Qualitative Analysis

This study took a mixed-methods approach. Quantitative survey questions were analysed in R 4.5.1 [72], using generalised linear models (GLMs). As we were interested in the effect of workshops on various measures, workshop attendance was the explanatory variable in all models. We ran models with the following response variables: (1) number of correct species identifications from photos, (2) number of species correctly identified as harmless vs. dangerous from photos, (3) number of those species incorrectly identified as harmless from photos, (4) number of those species incorrectly identified as dangerous from photos, and (5) the perceived mortality rate for untreated snakebites (recorded as a percentage and converted to proportion for analysis). Except for the last model (of perceived mortality rate), which was fitted with a binomial error distribution, all others were fitted with a Poisson error distribution, as they all model count data. In all cases, workshop attendees were set as the reference level, but we obtained *p*-values (and other parameters) for the difference between non-attendees who had vs. had not spoken to an attendee by re-running each model with attendees who had spoken to an attendee as the reference level. All Poisson models were checked for overdispersal, but there was no evidence of this in any of the models.

The quantitative aspects of the interviews were collated and summarised but were not analysed statistically, due to the small sample size. To enrich quantitative data, we also provided a narrative summary of workshop observation notes and interview data. Key themes and sub-themes in relation to Knowledge, Attitude, and Practices were identified by team members 2, 4, and 6 and were confirmed across the research team. The data used for each section of the results is outlined in Table S8.

## Figures and Tables

**Table 1:** Attitudes of interviewees towards snakes.

Attitude Valence	Workshop Non-Attendees (n = 4)	Workshop Non-Attendees Who Had Spoken to an Attendee (n = 7)	Workshop Attendees (n = 10)
Positive	0	0	6 (60%)
Mixed	2 (50%)	1 (14%)	4 (40%)
Negative	2 (50%)	6 (86%)	0
**Comments:**	All reported negative views of snakes due to the fear of bites/danger/death, but two also named potential benefits of snakes (tourism and skins).	All reported negative views of snakes and one of these participants reported attempting to kill snakes if they had the opportunity. Another participant reported negative feelings, but the information they received from an attendee resulted in them thinking they can now avoid snakes. One person said that their feelings are mixed but they mostly dislike them.	Six of the attendees report positive views of snakes (for example reporting that they have ‘no problems’ with snakes or that they ‘love them’). Four report fear of snakes but also acknowledge that they are now equipped with knowledge to coexist with them. All 10 attendees reported changes in attitude since the workshop, with the 5 that reported that they used to hit or kill snakes unanimously stating that they no longer do.

**Table 2:** Community perceptions regarding the beneficial qualities of snakes identified during interviews.

Benefit of Snakes	Workshop Non-Attendees (n = 4)	Workshop Non-Attendees Who Had Spoken to an Attendee (n = 7)	Workshop Attendees (n = 10)
None	1 (25%)	3 (43%)	0
Making drums	2 (50%)	1 (14%)	5 (50%)
Making shoes, belts, bags	1 (25%)	2 (29%)	7 (70%)
Rat or vermin control	1 (25%)	0	5 (50%)
Medicine (generally)	0	2 (29%)	1 (10%)
Medicine (anti-venom specifically)	0	2 (29%)	2 (20%)
Tourism	2 (50%)	1 (14%)	4 (40%)
Research/study	0	1 (14%)	4 (40%)
Food	1 (for dogs) (25%)	0	3 (30%) (although not in this community)
For pleasure	0	1 (14%)	0
Can grow mushrooms	0	1 (14%)	0

**Table 3:** Summary of snakebite experiences, fatalities, and estimation of snakebite fatality from survey data (questions 2–5).

	Overall (n = 76)	Workshop Non-Attendees (n = 43)	Workshop Non-Attendees Who Had Spoken to an Attendee (n = 8)	Workshop Attendees (n = 25)
**Mean and range of bites experienced**	Total = 178	Total = 87	Total = 16	Total = 75
	Mean = 2.3 per person	Mean = 1.9	Mean = 2.0	Mean = 3.0
	Range = 1–6 per person	Range = 1–6 pp	Range = 1–3 pp	Range = 1–6 pp
**Bites resulting in death**	Total = 6	Total = 2	Total = 1	Total = 3
	Mean = 0.07 per person	Mean = 0.04	Mean = 0.125	Mean = 0.12
	Range = 0–1 per person	Range = 0–1 pp	Range = 0–1 pp	Range = 0–1 pp
	Observed fatality rate = 3.37%	Observed fatality rate = 2.30%	Observed fatality rate = 6.25%	Observed fatality rate = 4%
**Perception of snakebite fatality resulting from dangerous snakes**	Mean assumption is that 71% of bites result in death	Mean assumption is that 89% of snakebites result in death	Mean assumption is that 89% of snakebites result in death	Mean assumption is that 33% of snakebites result in death
	Range 0–100%	Range 20–100%	Range 72–100%	Range 0–100%

**Table 4:** Circumstances of bites reported by interviewees.

Circumstances of Bite	Number of Occasions Reported, Split by the Gender of Bitten Person (Where This Information Was Provided)
In the home at a table	1 male 1 female
In bed	1 male 3 female
Manual work in garden or plantation	3 male 5 female (and a further female was spat on in the face by a cobra—no bite) 2 unknown
Outside at night (most reported as stepping on the snake in the dark)	5 male 3 female 2 unknown
Stepped on snake (unclear whether in the day or night)	1 male 1 female
Trying to kill a snake	2 male

**Table 5:** Personal treatment of snakebite suggestions identified by survey data (Question 7).

Treatment Option	Workshop Non-Attendees (n = 43)	Workshop Non-Attendees Who Had Spoken to an Attendee (n = 8)	Workshop Attendees (n = 25)
Seek hospital or other formal healthcare treatment	24 (56%)	3 (38%)	19 (76%)
Kill the snake	21 (49%)	3 (38%)	0
Drink old ladies’ urine	15 (35%)	3 (38%)	3 (12%)
Tie a cloth or similar item around the wound	12 (28%)	1 (13%)	3 (12%)
Run away	8 (19%)	1 (13%)	0
Clean the wound	8 (19%)	1 (13%)	0
Use snake beans	5 (12%)	1 (13%)	2 (8%)
Call for help	5 (12%)	1 (13%)	0
Cut around the wound	4 (9%)	0	0
Use a black stone	2 (5%)	0	0
Remain still	1 (2%)	0	0
Apply a coin	0	0	1 (4%)

**Table 6:** Personal practices to prevent snakebites identified by interview data.

Prevention Method	Workshop Non-Attendees (n = 4)	Workshop Non-Attendees Who Had Spoken to an Attendee (n = 7)	Workshop Attendees (n = 10)
Cutting grass and other vegetation short in the area surrounding house	1 (25%)	3 (43%)	6 (60%)
Keeping area in and around house tidy and clear of stones, bricks, log-piles or other objects that snakes may use to shelter	2 (50%)	2 (29%)	6 (60%)
Using lights when walking at night	0	2 (29%)	5 (50%)
Using mosquito nets	1 (25%)	0	4 (40%)
Ensure bed is raised off the ground	0	0	1 (10%)
Burning fish bones or aromatic vegetation around the house as repellent	1 (25%)	0	0
Planting tobacco around house as a repellent	2 (50%)	0	0
Blocking gaps in walls or doorways of houses	0	3 (43%)	6 (60%)
Remove rats from house	1 (25%)	0	0
Wear closed footwear such as boots when walking outside	0	2 (29%)	2 (20%)
None identified	0	2 (29%)	0

**Table 7:** Responses to coming across snakes based on interview data, separated by workshop attendance. Note that the middle column does not add up to 7 interviewees, as two of these said they would either kill the snake or run away and so both responses were counted here.

Response	Non-Attendees (n = 4)	Non-Attendees Who Had Spoken to an Attendee (n = 7)	Attendees (n = 10)
Kill the snake	3 (75%)	5 (71%)	0
Give the snake space and let it go away on its own	1 (25%)	4 (57%)	10 (100%)

**Table 8:** Background of persons involved in workshop content design.

Design and Deliver Team ID	Occupation	Gender	Country
1	Wildlife research project manager	M	Uganda
2	Zoologist	F	UK
3	Teacher	M	Uganda
4	Herpetologist	M	UK
5	Herpetologist	M	Uganda
6	Community Engagement specialist and biological anthropologist	F	UK

## Data Availability

The data presented in this study are available upon request from the corresponding author in summarised form, where possible, for reasons of privacy.

## Supplementary Materials

The following supporting information can be downloaded at: https://www.mdpi.com/article/10.3390/toxins18020078/s1, Tables S1–S8; Semi-structured interview questions; Snakes and snakebite survey.

## Abbreviation

The following abbreviation is used in this manuscript:

KAP	Knowledge, attitudes, and practices

## References

[1] Gutiérrez J.M., Calvete J.J., Habib A.G., Harrison R.A., Williams D.J., Warrell D.A. (2017). Snakebite envenoming. Nat. Rev. Dis. Primers.

[2] World Health Organization Neglected Tropical Diseases. https://www.who.int/health-topics/neglected-tropical-diseases.

[3] Gutiérrez J.M., Mackessy S.P. (2021). Snakebite envenomation as a Neglected Tropical Disease: New impetus for confronting an old scourge. Handbook of Venoms and Toxins of Reptiles.

[4] Martins S.B., Bolon I., Alcoba G., Ochoa C., Torgerson P., Sharma S.K., Ray N., Chappuis F., de Castañeda R.R. (2022). Assessment of the effect of snakebite on health and socioeconomic factors using a One Health perspective in the Terai region of Nepal: A cross-sectional study. Lancet Glob. Health.

[5] Bolon I., Finat M., Herrera M., Nickerson A., Grace D., Schütte S., Martins S.B., de Castañeda R.R. (2019). Snakebite in domestic animals: First global scoping review. Prev. Vet. Med..

[6] Bolon I., Martins S.B., Ochoa C., Alcoba G., Herrera M., Boyogueno H.M.B., Sharma B.K., Subedi M., Shah B., Wanda F. (2021). What is the impact of snakebite envenoming on domestic animals? A nation-wide community-based study in Nepal and Cameroon. Toxicon X.

[7] Martins S.B., Bolon I., Chappuis F., Ray N., Alcoba G., Ochoa C., Sharma S.K., Nkwescheu A.S., Wanda F., Durso A.M. (2019). Snakebite and its impact in rural communities: The need for a One Health approach. PLoS Negl. Trop. Dis..

[8] Farooq H., Geldmann J. (2024). The fear factor—Snakes in Africa might be at an alarming extinction risk. Conserv. Lett..

[9] The IUCN Red List of Threatened Species. https://www.iucnredlist.org.

[10] Halilu S., Iliyasu G., Hamza M., Chippaux J.-P., Kuznik A., Habib A.G. (2019). Snakebite burden in sub-Saharan Africa: Estimates from 41 countries. Toxicon.

[11] Farooq H., Bero C., Guilengue Y., Elias C., Massingue Y., Mucopote I., Nanvonamuquitxo C., Marais J., Faurby S., Antonelli A. (2022). Snakebite incidence in rural sub-Saharan Africa might be severely underestimated. Toxicon.

[12] Ddamulira J.B.M., Kasasa S., Kizito S., Mubangizi A., Kyaligonza J., Ronald S., Ntulume J. (2021). The Burden of Snakebite and Snakebite Envenoming in Uganda: A Community Survey and Facility Audit.

[13] Kasturiratne A., Wickremasinghe A.R., De Silva N., Gunawardena N.K., Pathmeswaran A., Premaratna R., Savioli L., Lalloo D.G., De Silva H.J. (2008). The global burden of snakebite: A literature analysis and modelling based on regional estimates of envenoming and deaths. PLoS Med..

[14] Chippaux J.-P., Mackessy S.P. (2021). Snakebite in Africa: Current situation and urgent needs. Handbook of Venoms and Toxins of Reptiles.

[15] World Health Organization Snakebite Data Information Portal. https://snbdatainfo.who.int/?page=Cases.

[16] Uetz P., Etzold T. (1996). The EMBL/EBI Reptile Database. Herpetol. Rev..

[17] WHO (2017). Expert Committee on Biological Standardization. Guidelines for the Production, Control and Regulation of Snake Antivenom Immunoglobulins.

[18] Longbottom J., Shearer F.M., Devine M., Alcoba G., Chappuis F., Weiss D.J., Ray S.E., Ray N., Warrell D.A., de Castañeda R.R. (1996). Vulnerability to snakebite envenoming: A global mapping of hotspots. Lancet.

[19] Hughes D.F., Behangana M. (2025). How many reptile and amphibian species are in Uganda, and why it matters for global biodiversity conservation. PeerJ.

[20] Tusabe J., Muhoozi M., Kajungu D., Mukose A., Kasasa S., Sebina Kibira S.P. (2025). Knowledge, perceptions and healthcare practices of communities for management of snakebites in Kamuli District, Eastern Uganda. Trans. R. Soc. Trop. Med. Hyg..

[21] Wangoda R., Watmon B., Kisige M. (2004). Snakebite management: Experiences from Gulu Regional Hospital Uganda. East. Cent. Afr. J. Surg..

[22] Wafula S.T., Mugume I.B., Namakula L.N., Nalugya A., Naggayi V., Walekhwa A.W., Musoke D. (2023). Healthcare practitioners’ knowledge of snakebite management and associated factors in high-burden, low-resource settings in Uganda. Trans. R. Soc. Trop. Med. Hyg..

[23] Wafula S.T., Namakula L.N., Ninsiima L.R., Ssekamatte N.K., Walekhwa A.W., Mugume I.B., Musoke D. (2023). Barriers and opportunities for improving management of snakebites: Perspectives of healthcare workers in Northern Uganda. PLoS ONE.

[24] Nanyonga S.M., Matafwali S.K., Kibira D., Kitutu F.E. (2025). Treatment and treatment outcomes of snakebite envenoming in Uganda: A retrospective analysis. Trans. R. Soc. Trop. Med. Hyg..

[25] Gutiérrez J.M., Williams D., Fan H.W., Warrell D.A. (2010). Snakebite envenoming from a global perspective: Towards an integrated approach. Toxicon.

[26] Abdullahi S.A., Habib A.G., Hussaini N. (2021). Control of snakebite envenoming: A mathematical modeling study. PLoS Negl. Trop. Dis..

[27] Moos B., Williams D., Bolon I., Mupfasoni D., Abela-Ridder B., de Castaneda R.R. (2021). A scoping review of current practices on community engagement in rural East Africa: Recommendations for snakebite envenoming. Toxicon X.

[28] Rodrigo C., Gnanathasan A. (2024). Lack of controlled studies on snakebite prevention: A rapid review. Trans. R. Soc. Trop. Med. Hyg..

[29] Michie S., van Stralen M.M., West R. (2011). The behaviour change wheel: A new method for characterising and designing behaviour change interventions. Implement. Sci..

[30] Cook P.A., Bellis M.A. (2001). Knowing the risk: Relationships between risk behaviour and health knowledge. Public Health.

[31] Glasman L.R., Albarracín D. (2006). Forming attitudes that predict future behavior: A meta-analysis of the attitude-behavior relation. Psychol. Bull..

[32] Carter H., Glaudas X., Whitaker R., Chandrasekharun G., Hockings K., Nuno A. (2024). Venomous snakebites: Exploring social barriers and opportunities for the adoption of prevention measures. Conserv. Sci. Pract..

[33] Mitchell J., Cooke P., Ahorlu C., Arjyal A., Baral S., Carter L., Dasgupta R., Fieroze F., Fonseca-Braga M., Huque R. (2022). Community engagement: The key to tackling Antimicrobial Resistance (AMR) across a One Health context?. Glob. Public Health.

[34] Henley P., Igihozo G., Wotton L. (2021). One Health approaches require community engagement, education, and international collaborations—A lesson from Rwanda. Nat. Med..

[35] Galarde-López M., Quiroz-Rocha G.F., Candanosa-Aranda I.E., Soberanis-Ramos O., García-García L. (2022). Community engagement in the diagnosis and control of a bovine paralytic rabies outbreak in two rural communities of Mexico. J. Agromed..

[36] Nkansah-Dwamena E. (2023). Lessons learned from community engagement and participation in fostering coexistence and minimizing human-wildlife conflict in Ghana. Trees For. People.

[37] Schiavo R. (2021). What is true community engagement and why it matters (now more than ever). J. Commun. Healthc..

[38] King R., Hicks J., Rassi C., Shafique M., Barua D., Bhowmik P., Das M., Elsey H., Questa K., Fieroze F. (2020). A process for developing a sustainable and scalable approach to community engagement: Community dialogue approach for addressing the drivers of antibiotic resistance in Bangladesh. BMC Public. Health.

[39] Ten Have N., Ooms G.I., Waldmann B., Reed T. (2023). Barriers and enablers of community engagement practices for the prevention of snakebite envenoming in South Asia: A qualitative exploratory study. Toxicon X.

[40] Vaiyapuri S., Kadam P., Chandrasekharuni G., Oliveira I.S., Senthilkumaran S., Salim A., Patel K., Sachett J.D.A.G., Pucca M.B. (2023). Multifaceted community health education programs as powerful tools to mitigate snakebite-induced deaths, disabilities, and socioeconomic burden. Toxicon X.

[41] Tolo C.U., Kahwa I., Nuwagira U., Weisheit A., Ikiriza H. (2023). Medicinal plants used in treatment of various diseases in the Rwenzori Region, Western Uganda. Ethnobot. Res. Appl..

[42] Samuel S.P., Chinnaraju S., Williams H.F., Pichamuthu E., Subharao M., Vaiyapuri M., Arumugam S., Vaiyapuri R., Baksh M.F., Patel K. (2020). Venomous snakebites: Rapid action saves lives—A multifaceted community education programme increases awareness about snakes and snakebites among the rural population of Tamil Nadu, India. PLoS Negl. Trop. Dis..

[43] Wolfe A.K., Fleming P.A., Bateman P.W. (2020). What snake is that? Common Australian snake species are frequently misidentified or unidentified. Hum. Dimens. Wildl..

[44] Corbett S.W., Anderson B., Nelson B., Bush S., Hayes W.K., Cardwell M.D. (2005). Most lay people can correctly identify indigenous venomous snakes. Am. J. Emerg. Med..

[45] Morrison J.J., Pearn J.H., Nixon J., Covacevich J. (1983). Can Australians identify snakes?. Med. J. Aust..

[46] Landová E., Bakhshaliyeva N., Janovcová M., Peléšková Š., Suleymanova M., Polák J., Guliev A., Frynta D. (2018). Association between fear and beauty evaluation of snakes: Cross-cultural findings. Front. Psychol..

[47] Frynta D., Štolhoferová I., Elmi H.S.A., Janovcová M., Rudolfová V., Rexová K., Sommer D., Král D., Berti D.A., Landová E. (2025). Hooding cobras can get ahead of other snakes in the ability to evoke human fear. Sci. Nat..

[48] Duda R., Monteiro W.M., Giles-Vernick T. (2021). Integrating lay knowledge and practice into snakebite prevention and care in central Africa, a hotspot for envenomation. Toxicon X.

[49] Randler C., Zehender I. (2006). Effectiveness of reptile species identification-A comparison of dichotomous key with an identification book. Eurasia J. Math. Sci. Technol. Educ..

[50] Smith L.H. (2020). Selection mechanisms and their consequences: Understanding and addressing selection bias. Curr. Epidemiol. Rep..

[51] Henke S.E., Kahl S.S., Wester D.B., Perry G., Britton D. (2019). Efficacy of an online native snake identification search engine for public use. Hum. Wildl. Interact..

[52] Malhotra A., Wüster W., Owens J.B., Hodges C.W., Jesudasan A., Ch G., Kartik A., Christopher P., Louies J., Naik H. (2021). Promoting co-existence between humans and venomous snakes through increasing the herpetological knowledge base. Toxicon X.

[53] Western D., Nightingale D.M., Mose V.N., Sipitiek J.O., Kimiti K.S. (2019). Variability and change in Maasai views of wildlife and the implications for conservation. Hum. Ecol..

[54] Keener-Eck L.S., Morzillo A.T., Christoffel R.A. (2020). A comparison of wildlife value orientations and attitudes toward timber rattlesnakes (*Crotalus horridus*). Hum. Dimens. Wildl..

[55] Kansky R., Kidd M. (2024). Putting yourself in an animal’s shoes-empathy and intangible benefits drive tolerance towards wildlife in Namibian communal conservancies. Biol. Conserv..

[56] Ramesh C., Nehru P. (2019). Living with snakes in India: The intensifying health crisis over snakebites–challenges and way ahead. Asian J. Conserv. Biol..

[57] Larson K.L., Clark J.A.G., Bateman H.L., Enloe A., Hughes B. (2024). To kill or not to kill? Exploring normative beliefs and attitudes toward snakes. Biol. Conserv..

[58] Pandey D.P., Subedi G., Sapkota S., Dangol D.R., Devkota N.R. (2023). Attitudes, knowledge and practices of traditional snakebite healers in Nepal: Implications for prevention and control of snakebite. Trans. R. Soc. Trop. Med. Hyg..

[59] Pandey D.P., Subedi G., Devkota K., Goode M. (2016). Public perceptions of snakes and snakebite management: Implications for conservation and human health in southern Nepal. J. Ethnobiol. Ethnomed..

[60] Bruder J., Burakowski L.M., Park T., Al-Haddad R., Al-Hemaidi S., Al-Korbi A., Al-Naimi A. (2022). Cross-cultural awareness and attitudes toward threatened animal species. Front. Psychol..

[61] Maduwage K., Gamage S.K., Gutiérrez J.M. (2024). First aid and pre-hospital practices in snakebite victims: The persistent use of harmful interventions. Toxicon.

[62] Fry B.G. (2018). Snakebite: When the human touch becomes a bad touch. Toxins.

[63] Gum B., Opoke R., Akwongo B., Oloya B., Omony J.B., Opiro R., Andama M., Anywar G., Malinga G.M. (2024). An ethnobotanical survey of plant species used for medicinal purposes in Amuru district, northern Uganda. Ethnobot. Res. Appl..

[64] Newman W.J., Moran N.F., Theakston R.D.G., Warrell D.A., Wilkinson D. (1997). Traditional treatments for snake bite in a rural African community. Ann. Trop. Med. Parasitol..

[65] Chuat M., Alcoba G., Eyong J., Wanda F., Comte E., Nkwescheu A., Chappuis F., Hudelson P. (2021). Dealing with snakebite in rural Cameroon: A qualitative investigation among victims and traditional healers. Toxicon X.

[66] Iddi S., Justin J., Hamasaki K., Konje E.T., Kongola G.W. (2022). Assessment of snakebite management practices at Meserani Juu in Monduli District, Northern Tanzania. PLoS ONE.

[67] Global Snakebite Initiative Prevention Videos. https://www.globalsnakebite.org/prevention-videos/.

[68] Treves A., Wallace R.B., Naughton-Treves L., Morales A. (2006). Co-managing human–wildlife conflicts: A review. Hum. Dimens. Wildl..

[69] Nyhus P.J. (2016). Human–wildlife conflict and coexistence. Annu. Rev. Environ. Resour..

[70] Dickman A.J. (2010). Complexities of conflict: The importance of considering social factors for effectively resolving human–wildlife conflict. Anim. Conserv..

[71] World Health Organization Snakebite Envenoming. https://www.who.int/health-topics/snakebite.

[72] R Core Team (2025). R: A Language and Environment for Statistical Computing.

