# Supplementary material for: A Community Engagement Approach to Snakebite Prevention in Rural Uganda: Exploring Knowledge, Attitudes, and Practices
Source: Toxins (Basel). 2026 Feb 2;18(2):78. doi: 10.3390/toxins18020078 (PMC12944939; doi:10.3390/toxins18020078)
Supplement: Supplementary file 1 [file toxins-18-00078-s001.zip › toxins-4083118-supplementary.pdf]

Supplementary Materials: A Community Engagement Approach to Snakebite Prevention in Rural Uganda: Exploring Knowledge, Attitudes, and Practices

Kevin Arbuckle, Yowasi Byaruhanga, Hazel J. Nichols, Cris M. Kaseke, Francis Mwanguhya, and Jessica Mitchell

Table S1. Snake species correctly identified from photographs by 76 survey responses (survey question 8).

Snake name	Workshop non-attendees (n = 43) able to identify the snake	Workshop non-attendees who had spoken to an attendee (n = 8) able to identify the snake	Workshop attendees (n = 25) able to identify the snake
Brown house snake (*Boaedon fuliginosus*)	33 (77%)	4 (50%)	19 (76%)
Brown forest cobra (defensive posture) (*Naja subfulva*)	32 (74.%)	7 (88%)	24 (96%)
Puff adder (*Bitis arietans*)	0	1 (13%)	18 (72%)
Boomslang (*Dispholidus typus*)	0	0	0
Brown forest cobra (non-defensive posture) (*Naja subfulva*)	1 (2.%)	0	0
White-lipped snake (*Crotaphopeltis hotamboeia*)	0	0	10 (40%)
Burrowing asp (*Atractaspis irregularis*)	0	0	1 (4%)
Bush viper (*Atheris hispida*)	0	0	7 (28%)
Green bush snake (*Philothamnus semivariegatus*)	0	0	11 (44%)
Rock python (*Python sebae*)	5 (12%)	3 (38%)	17 (68%)
Olive sand snake (*Psammophis mossambicus*)	0	0	10 (40%)
Jameson's mamba (*Dendroaspis jamesoni*)	0	0	2 (8%)

Table S2. Commonly identifiable snakes and their descriptions from 21 interviews. We have translated local names into English names and provided the corresponding scientific name based on our best estimate from the descriptions of the snakes provided by interviewees.

Local Name	English Name	Workshop non-attendees (n = 4)	Workshop non-attendees who had spoken to an attendee (n = 7)	Workshop attendees (n = 10)	Common descriptions
Enchwera	Black Spitting Cobra (*Naja nigricollis*)	3 (75%)	6 (86%)	7 (70%)	Black (sometimes with yellow), large, and fast moving. Raises up and forms a hood when disturbed.
Empiri	Puff Adder (*Bitis arietans*)	1 (25%)	3 (43%)	5 (50%)	Short but big. Slow and with spots (sometimes described as black and yellow).
Enzaramire	Rock Python (*Python sebae*)	2 (50%)	3 (43%)	5 (50%)	Yellowish and 'Spotty' - interviewees referred to patterned items of clothing/handbags to describe the appearance. Tough and commonly referred to as capable of eating humans and livestock. Large and many people referred to the usage of its skin in drums and other artefacts (shoes, bags etc.)
Ekirya mbeba	'Rat Eater' (*Boaedon* sp.)	3 (75%)	4 (57%)	6 (60%)	Black and shiny. Found within the house where it eats rats and other vermin
Enyarubabi	'Green Snake'[1] (*Philothamnus* sp.)	3 (75%)	4 (57%)	9 (90%)	Thin and 'slippery' but also to move quickly. Bright green and small (but long), often described as looking like grass or leaves. Lives in trees.

[1] Green Snake could plausibly also refer to a Boomslang (*Dispholidus*) either deliberately or due to confusion of the species, but typical description better matched *Philothamnus* sp.

Table S3. Snake species correctly identified as harmless or dangerous to humans from photographs in 76 responses (survey question 8).

Snake name	Risk category	Workshop non-attendees (n = 43) correctly assessed risk	Workshop non-attendees who had spoken to an attendee (n = 8) correctly assessed risk	Workshop attendees (n = 25) correctly assessed risk
Brown house snake (*Boaedon fuliginosus*)	Harmless	39 (91%)	7 (88%)	21 (84%)
Brown forest cobra (defensive posture) (*Naja subfulva*)	Dangerous	41 (95%)	8 (100%)	25 (100%)
Puff adder (*Bitis arietans*)	Dangerous	40 (93%)	8 (100%)	23 (92%)
Boomslang (*Dispholidus typus*)	Dangerous	38 (88%)	6 (75%)	3 (12%)
Brown forest cobra (non-defensive posture) (*Naja subfulva*)	Dangerous	42 (98%)	7 (88%)	9 (36%)
White-lipped snake (*Crotaphopeltis hotamboeia*)	Harmless	1 (2%)	2 (25%)	12 (48%)
Burrowing asp (*Atractaspis irregularis*)	Dangerous	39 (91%)	4 (50%)	9 (36%)
Bush viper (*Atheris hispida*)	Dangerous	41 (95%)	7 (88%)	19 (76%)
Green bush snake (*Philothamnus semivariegatus*)	Harmless	2 (5%)	0	15 (60%)
Rock python (*Python sebae*)	Dangerous	40 (93%)	8 (100%)	22 (88%)
Olive sand snake (*Psammophis mossambicus*)	Harmless	0	1 (13%)	11 (44%)
Jameson's mamba (*Dendroaspis jamesoni*)	Dangerous	39 (91%)	5 (63%)	17 (68%)

Table S4. Personal treatment of snakebite suggestions identified by interview data.

**Treatment option**	**Workshop non-attendees (n = 4)**	**Workshop non-attendees who had spoken to an attendee (n = 7)**	**Workshop attendees (n = 10)**
Seek hospital or other formal healthcare treatment	3 (75%)	4 (57%)	10 (100%)
Tie a cloth or similar item around the wound	3 (75%)	2 (29%)	0
Use snake beans	2 (50%)	0	0
Keep calm	0	0	4 (40%)
Local medication (unspecified) from traditional healer	0	3 (43%)	0

Table S5. Community norms in responses to snakebite identified by survey data (Question 6).

**Treatment option**	**Workshop non-attendees (n = 43)**	**Workshop non-attendees who had spoken to an attendee (n = 8)**	**Workshop attendees (n = 25)**
Seek hospital or other formal healthcare treatment	0	0	18 (72%)
Drink old ladies' urine	24 (56%)	7 (88%)	7 (28%)
Tie a cloth or similar item around the wound	20 (47%)	6 (75%)	7 (28%)
Use snake beans	22 (51%)	5 (63%)	12 (48%)
Cut around the wound	7 (16%)	2 (25%)	1 (4%)
Use a Black Stone	20 (47%)	2 (25%)	3 (12%)
Remain still	8 (19%)	0	0
Drink palm oil	12 (28%)	1 (13%)	2 (8%)
Faeces	1 (2%)	0	0

Table S6. Practices in the wider community (that others do) to prevent snakebite, as identified by interview data.

Prevention method	Workshop non-attendees (n = 4)	Workshop non-attendees who had spoken to an attendee (n = 7)	Workshop attendees (n = 10)
Cutting grass and other vegetation short in the area surrounding house	0	1 (14%)	3 (30%)
Keeping area in and around house tidy and clear of stones, bricks, log-piles or other objects that snakes may use to shelter	0	0	3 (30%)
Using lights when walking at night	1 (25%)	1 (14%)	1 (10%)
Burning fish bones or aromatic vegetation around the house as repellent	1 (25%)	0	1 (10%)
Spreading paraffin as a repellent	2 (50%)	0	0
Blocking gaps in walls or doorways of houses	1 (25%)	0	2 (20%)
Leave uncovered buckets of urine in bedrooms for snake to drink (as they are then believed to lose the will to bite)	1 (25%)	0	0
Wear closed footwear such as boots when walking outside	0	1 (14%)	0
Killing snakes that are seen	0	1 (14%)	2 (20%)
Leaving alone any snakes that are seen	0	0	1 (10%)
None identified (may be none in addition to those used personally)	1 (25%)	4 (57%)	3 (30%)

Table S7. Suggestions from community members during workshop of current methods ('doing now') and acceptable changes ('could do') that would help to prevent snakebite in the area. Numbers are how many of the 7 workshop sessions each method was reported as either being done currently or an acceptable change.

**Prevention method**	**Currently doing (at time of workshops)**	**Could acceptably do in future (for at least some people)**
Cutting grass and other vegetation short in the area surrounding house	6	3
Keeping area in and around house tidy and clear of stones, bricks, log-piles or other objects that snakes may use to shelter	7	7
Using lights at night	3	7
Burning fish bones or aromatic vegetation around the house as repellent	2	1
Spreading paraffin as a repellent	1	0
Using mosquito nets	5	5
Blocking gaps in walls or doorways of houses (including having a raised step at the doorway)	6	5
Ensure bed is raised off the ground	4	1
Wear closed footwear such as boots when walking outside	5	6
Stepping heavily on ground when walking in high risk areas	1	1
Avoid rats in house (e.g. trapping and killing)	1	2
Leaving alone any snakes that are seen	1	1
Cover water sources and remove stagnant water	2	1
Carefully checking for snakes before putting hands or other body parts where they may hide, or avoid going near such places unnecessarily	4	4

Table S8. Datasets used to derive information about Knowledge, Attitude and Practices in relation to snakes and snakebite.

Theme	Sub-theme	Dataset	Specific section/question
**Knowledge**	Snake species	Survey	Question 8
		Interview	Question 3
	Snake risk assessment	Workshop	Section 3
		Survey	Question 8
**Attitude**	General feelings on snakes	Workshop	Sections 1 - 3
		Interview	Question 4
	Benefits of snakes	Workshop	Section 2
		Interview	Question 5
	Recollections of snakebite experiences	Survey	Questions 3 and 4
		Interview	Question 9
	Perception of snakebit threat	Survey	Questions 2 - 5
**Practice**	Community norms in snakebite treatment	Survey	Question 6
		Interview	Question 11
	Personal snake (bite) response	Survey	Question 7
	Snakebite prevention in the community	Interview	Questions 10, 12 and 13
**Other**	Key learnings from the workshop	Interview	Question 1
	Knowledge diffusion from workshops	Interview	Question 1

Snakes and Snakebite – Interview Questions

Note that the interviews are intended to give us an understanding of current knowledge, beliefs, attitudes, and experiences of snakes and snakebites, in the community. They will also be fairly open in the sense that, although some structuring is provided by the questions below, participants will be encouraged to discuss views openly and without artificial constraints. The format is a one-to-one interview with the researchers to talk about the participant's perceptions and experiences of snakes and snakebite, including any cultural practices and stories they are aware of. This will not be rushed and is quite an open format to give time and opportunity to talk about anything relevant to the subject, but we expect the interviews will be less than 1 hour.

Question 1: Did you attend one of the snakebite prevention workshops (if so, what did you learn from the workshop? Did you tell other people about what you learned? Who (family? Wider community? Etc.), or speak to anyone who did attend about it?

Question 2: How often do you see snakes around your community (and in what context – home? Work? Etc.)? What are the snakes doing when you see them?

Question 3: Do you know what kind they are? Can you describe them?

Question 4: What do you think about snakes (do you like them or not, and why)?

Question 5: Do you think snakes have any positive or useful qualities?

Question 6: Do you know any folktales, stories, or other tales involving snakes? Do you believe these?

Question 7: Is there anything you have been told about snakes that you don't believe? What are these things and where did you hear them (Family member? Community member? Stranger? Educator? Someone else?)?

Question 8: Do (any) snakes have a special cultural significance to your or your community? If so, which snakes and how are they embedded in your culture?

Question 9: Do you know anyone in your community who has been bitten by a snake? If so, how many people and what happened before, during and after the bites (please clarify if, or which bits, this information is what you witnessed or second-hand from someone else)?

Question 10: What would you do if you came across a snake? (if participant attended a workshop, ask if this has changed their answer to this question).

Question 11: What would you do if you or someone else was bitten by a snake (especially if you believed the snake to be dangerous)? (if participant attended a workshop, ask if this has changed their answer to this question).

Question 12: Is there anything you normally do as a way of avoiding snakes or preventing being bitten by them? Do you know of anything others do to prevent snakebite? (if participant attended a workshop, ask if this has changed their answer to this question).

Question 13: If participant attended workshop: can you remember any of the things we discussed to prevent snakebites in the workshop?

Question 14: We are very broadly interested in understanding local knowledge, culture, attitudes, and experiences of snakes and snakebite (especially but not only anything that might be relevant to helping to reduce snakebite). Is there anything else you think that we might be interested to know or would like to tell us?

Survey on Snakes and Snakebite

The aim of this survey is to let us understand the local context of snakebite, including knowledge and attitudes towards snakes as well as how common snakebite is in the area. Please answer all questions to the best of your ability, this is not a test and we are not collecting any personal data on you for this survey, we just want to understand the problem we are trying to address in our wider project aimed at reducing snakebite in the community. We don't expect long answers on this survey, so it shouldn't take a lot of time to complete: some questions have boxes for you to tick an option, others ask only for a number or a short response of a few sentences at most.

1. Did you attend the snakebite prevention workshops or speak to anyone who did about them?

Attended workshop ☐

Didn't attend but have spoken with someone who did ☐

Didn't attend and haven't spoken to anyone who did ☐

2. How would you rate your knowledge of the snakes in your local area?

Poor ☐

OK ☐

Good ☐

High ☐

3. How many snake bites are you aware of in your community, and over what time period is this answer referring to?

……………………………………………………………………………………………

4. Of the bites you are aware of, how many resulted in the death of the person bitten?

…………………………………………………………………………………………………

5. If a person is bitten by a dangerous snake and does not receive any treatment at all, how likely do you believe they are to die (please give answer as a percentage)?

…………………………………………………………………………………………………

6. What treatments have been given to people who have been bitten in the local area?

…………………………………………………………………………………………………

…………………………………………………………………………………………………

…………………………………………………………………………………………………

…………………………………………………………………………………………………

7. If you were bitten by a snake you believed was dangerous, what would you do?

…………………………………………………………………………………………………

…………………………………………………………………………………………………

…………………………………………………………………………………………………

……………………………………………………………………………………………………

8. Under each of the pictures on the next three pages there are three lines. In the top lines, please say whether you are familiar with this snake and recognise it. In the middle lines, please write the name that you call the snake in the picture (or say if you do not know the name or can't identify it). In the bottom lines, please say whether you believe the snake in the picture is a dangerous or a harmless snake.

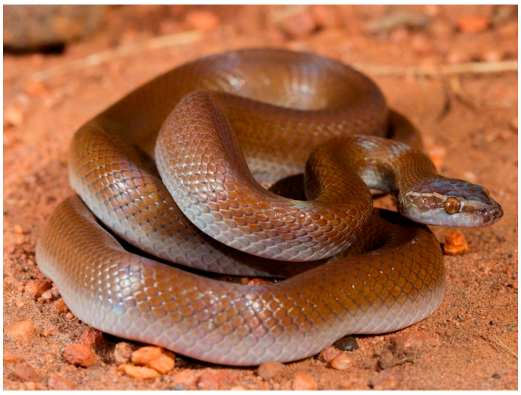

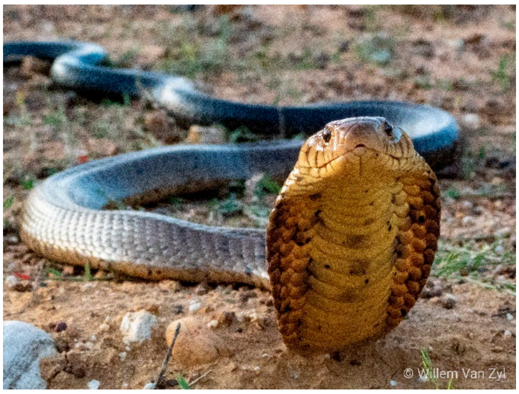

………………………………………………………………………….

………………………………………………………………………….

………………………………………………………………………….

………………………………………………………………………….

………………………………………………………………………….

………………………………………………………………………….

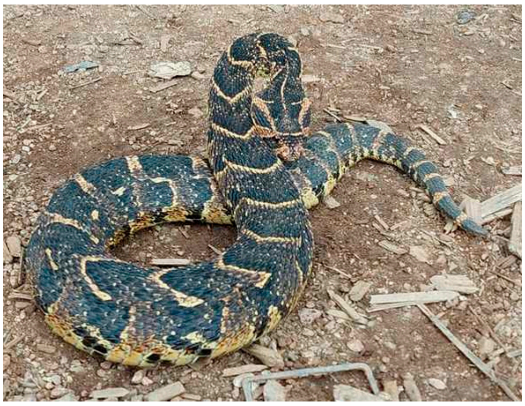

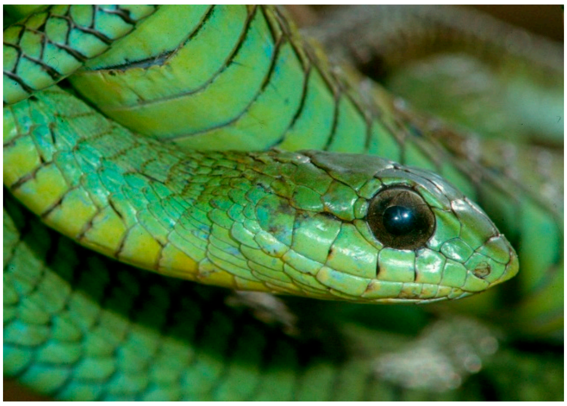

…………………………………………………………………………

…………………………………………………………………………

…………………………………………………………………………

…………………………………………………………………………

…………………………………………………………………………

…………………………………………………………………………

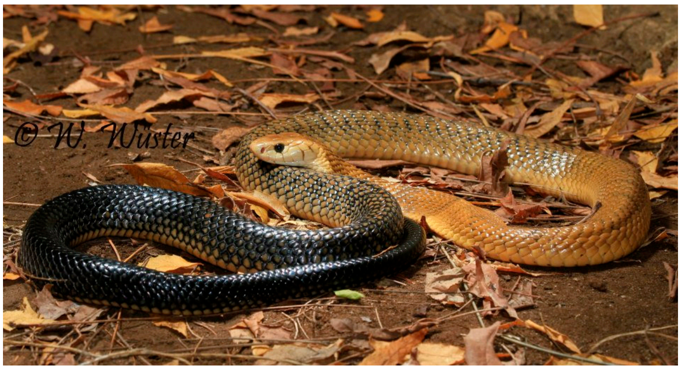
© W. Wüster

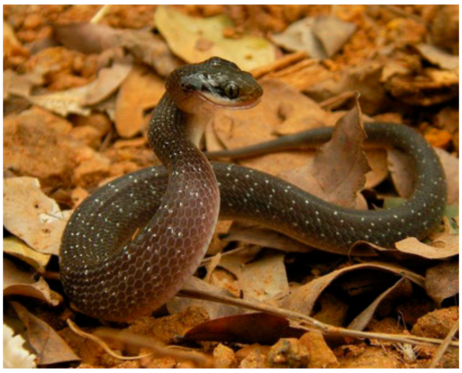

………………………………………………………………………………………………

…………………………………………………………………

………………………………………………………………………………………………

…………………………………………………………………

………………………………………………………………………………………………

…………………………………………………………………

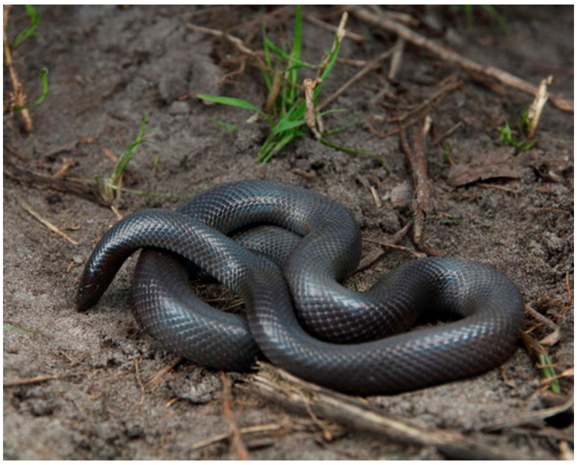

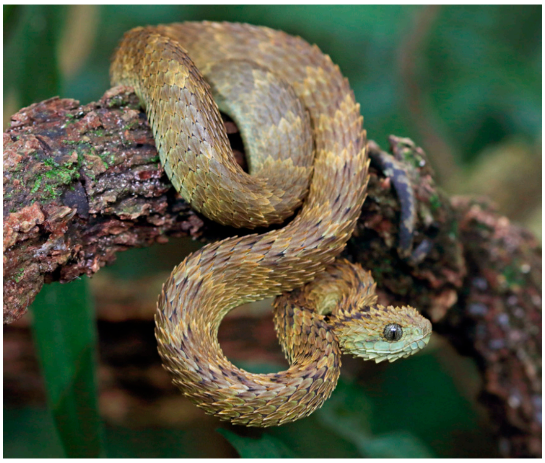

………………………………………………………………………..

………………………………………………………………………

………………………………………………………………………..

………………………………………………………………………

………………………………………………………………………..

………………………………………………………………………

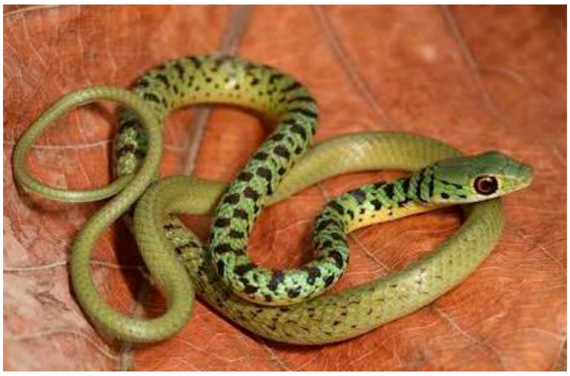

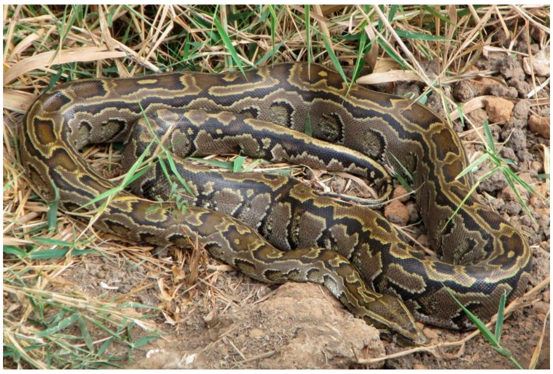

………………………………………………………………………..

……………………………………………………………………

……………………………………………………………………….

……………………………………………………………………

....

....

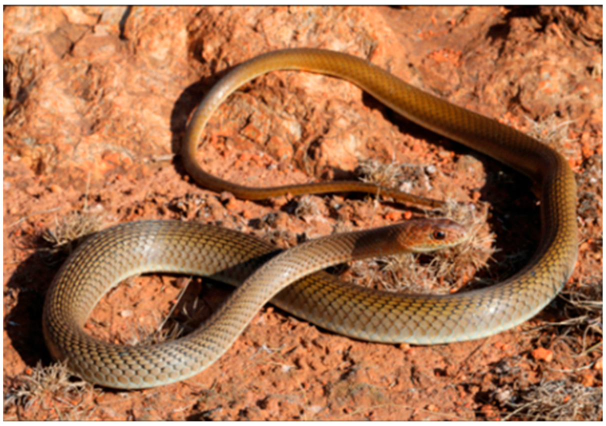

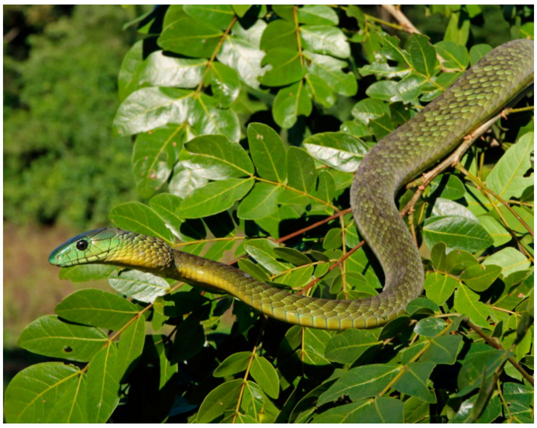

....

....

....

....

....

....
